# Deep conservation of cis-regulatory elements and chromatin organization in echinoderms uncover ancestral regulatory features of animal genomes

**DOI:** 10.1038/s41559-025-02941-y

**Published:** 2026-01-07

**Authors:** Marta S. Magri, Danila Voronov, Saoirse Foley, Pedro Manuel Martínez-García, Martin Franke, Gregory A. Cary, José M. Santos-Pereira, Claudia Cuomo, Manuel Fernández-Moreno, Marta Portela, Alejandro Gil-Galvez, Rafael D. Acemel, Periklis Paganos, Carolyn Ku, Jovana Ranđelović, Maria Lorenza Rusciano, Panos N. Firbas, José Luis Gómez-Skarmeta, Veronica F. Hinman, Maria Ina Arnone, Ignacio Maeso

**Affiliations:** 1 CABD (Centro Andaluz de Biología del Desarrollo), CSIC-Universidad Pablo de Olavide-Junta de Andalucía, Seville, Spain; 2 Department of Genetics, Microbiology and Statistics, University of Barcelona, Spain; 3 Institut de Recerca de la Biodiversitat (IRBio), Universitat de Barcelona, Spain; 4 Department of Biology and Evolution of Marine Organisms, Stazione Zoologica Anton Dohrn, Villa Comunale, 80121 Napoli, Italy; 5 Department of Biological Sciences, Carnegie Mellon University, Pittsburgh, PA 15213, United States; 6 Department of Biology, Marian University, Indianapolis, IN 46222, United States; 7 Departamento de Biología Celular, Facultad de Biología, Universidad de Sevilla, 41012 Sevilla, Spain.; 8 Instituto de Biomedicina de Sevilla (IBiS), Hospital Universitario Virgen del Rocío/CSIC/Universidad de Sevilla, 41013 Sevilla, Spain.; 9 Centre for Genomic Regulation, Barcelona, Spain; 10 Champalimaud Centre for the Unknown, Champalimaud Foundation, 1400-038 Lisbon, Portugal

## Abstract

Despite the growing abundance of sequenced animal genomes, we only have detailed knowledge of regulatory organization for a handful of lineages, particularly flies and vertebrates. These two taxa show contrasting trends in the molecular mechanisms of 3D chromatin organization and long-term evolutionary dynamics of cis-regulatory element (CREs) conservation. Here, we study the evolution and organization of the regulatory genome of echinoderms, a lineage whose phylogenetic position and relatively slow molecular evolution has proven particularly useful for evolutionary studies. We generated new reference genome assemblies for two species belonging to two different echinoderm classes: the purple sea urchin *Strongylocentrotus purpuratus* and the bat sea star *Patiria miniata* using PacBio and HiC data and characterize their 3D chromatin architecture. We show that these echinoderms have TAD-like domains that, like in flies, do not seem to be associated with CTCF motif orientation. We systematically profiled CREs during sea star and sea urchin development using ATAC-seq, comparing their regulatory logic and dynamics over multiple developmental stages. Finally, our analysis of sea urchin and sea star CRE evolution across multiple evolutionary distances and timescales showed several thousand elements conserved for hundreds of millions of years, revealing a vertebrate-like pattern of CRE evolution that probably constitutes an ancestral property of the regulatory evolution of animals.

## Introduction

The vast morphological disparity of animals rests on complex spatio-temporal regulation of gene expression. Unlike their unicellular relatives^1^, animal gene expression often depends on multiple cis-regulatory elements (CREs), which can act over very long genomic distances. However, different animal lineages achieve this precise regulation through different strategies, reflected in profound differences in their regulatory genome organization^2–8^. How have these diverse gene regulation strategies evolved in animals? Which are ancestral and which are derived? Although comparative functional genomics have begun to address these questions^6,7,9–12^, most lineages are still unsampled. Multiple works suggest that long-range regulation is ancestral to animals^13–18^, but the underlying molecular mechanisms differ markedly among lineages. In vertebrates, long-range interactions are mediated by loop extrusion, a mechanism dependent on cohesin and the zinc-finger protein CTCF. Chromatin loops are anchored by convergently oriented CTCF binding sites, a crucial configuration for these 3D structures^19–22^. However, in flies, genome contacts primarily depend on chromatin states and lineage-specific architectural proteins such as Cp190, BEAF-32 and Su(Hw)^23,24^. CRE conservation across deep evolutionary distances is also poorly understood. Most CREs show high turnover^25–30^ and in flies and nematodes CRE sequences are only conserved over short timescales^27,31–34^. In contrast, certain enhancers are extremely ancient, with a few examples predating the divergence of animal phyla (>600 mya)^35–37^. The biological and evolutionary significance of this extreme sequence conservation remains unresolved^38,39^, as widespread deep conservation of CREs has so far been observed only in vertebrates, with thousands of elements maintained across jawed vertebrates for over 450 million years^40–44^. The extent of deep CRE conservation in other lineages either remains unexplored^6,8,9,11,12^ or is impossible to assess due to their phylogenetic isolation^7,45^.

Echinoderms are well suited to fill this gap and to investigate the macroevolution of regulatory genome organization. Their extant species span a wide range of evolutionary timescales and retain several ancestral genomic features, including high conservation of developmental gene families^46^ and conserved macro- and microsynteny^13,16,47,48^. More importantly, several echinoderm species are powerful developmental models that have pioneered the study of gene regulatory networks (GRNs). Comparative analyses of GRNs, especially between sea stars and sea urchins, have revealed remarkable conservation of regulatory nodes, but also substantial network rearrangements after 500 Mya of evolution^49^. However, while both small-scale^50,51^ and genome-wide studies^52^ have investigated CRE evolution between closely related sea urchin species, genome-wide trends of CRE evolution across deeply diverged echinoderm and deuterostome lineages, and their 3D regulatory organization remain unexplored.

Here, we present a comprehensive study of the bat sea star and the purple sea urchin regulatory genomes and 3D chromatin organization, tracing their evolution across major echinoderm groups. Our results suggest that TAD-like chromatin structures are not ancestrally associated with a specific CTCF motif orientation and that deep conservation of CREs across hundreds of millions of years is far more widespread than previously recognized.

## Results

### New Bat Sea Star and Purple Sea Urchin Reference Genome Assemblies & Gene Annotations

**Extended Data Figure 1.**
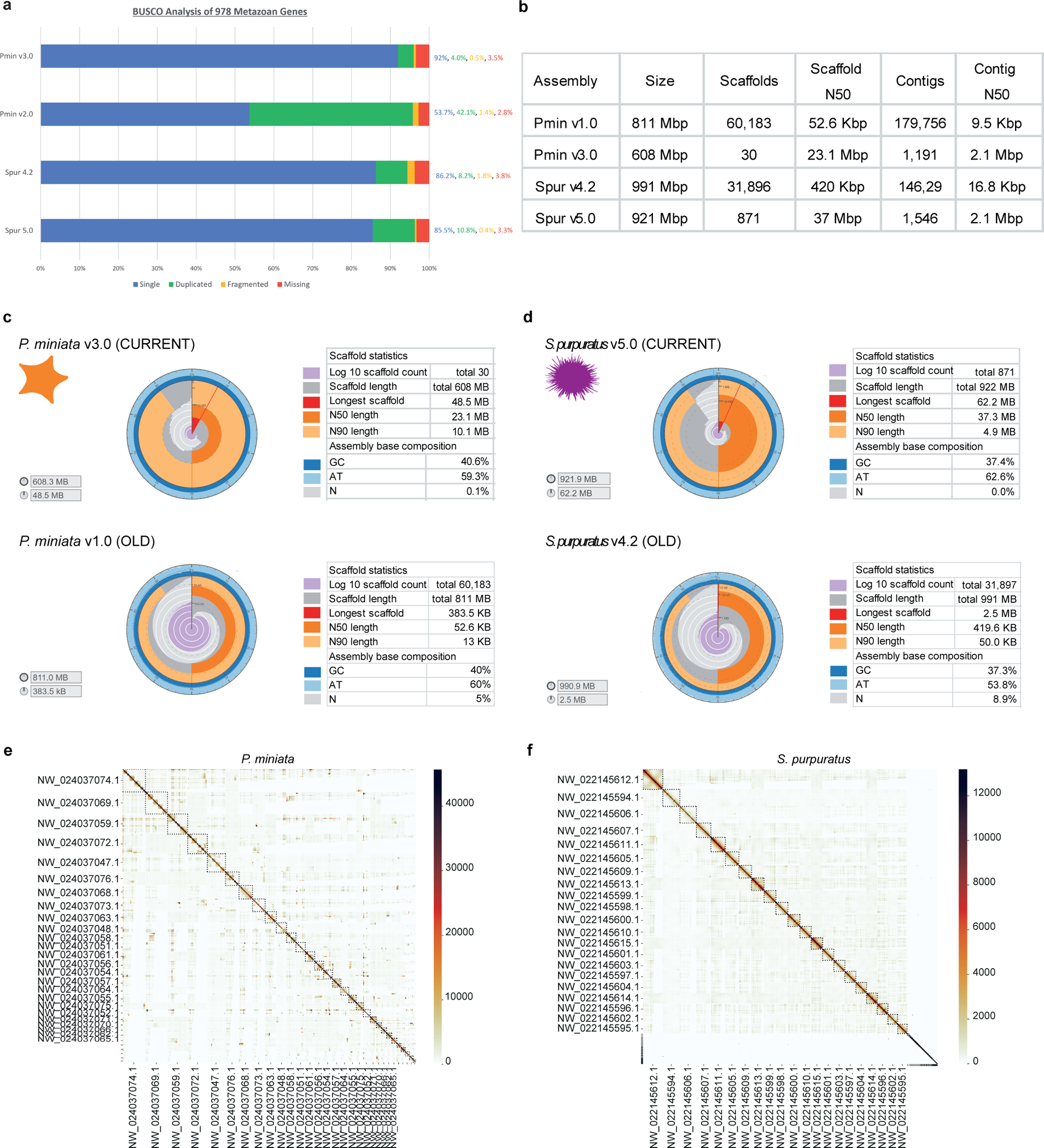
Comparison of current and previous assembly statistics: **a**, BUSCO statistics of current and previous genome assemblies. **b,** Assembly statistics of the two new genomes along with their older versions for comparison, generated with the assembly-stats perl package. **c, d,** Snailplots of general metrics (scaffold total length, scaffold count, longest scaffold, N50 and N90 values, and base compositions) of the new assemblies of the bat sea star (GCA_015706575.1) (c, upper panel) and purple sea urchin (GCA_000002235.4) (d, upper panel) versus those of previous assemblies (lower panels). **e, f,** HiC genome-wide contact maps of the bat sea star (e) and purple sea urchin (f) assemblies.

**Figure 1:**
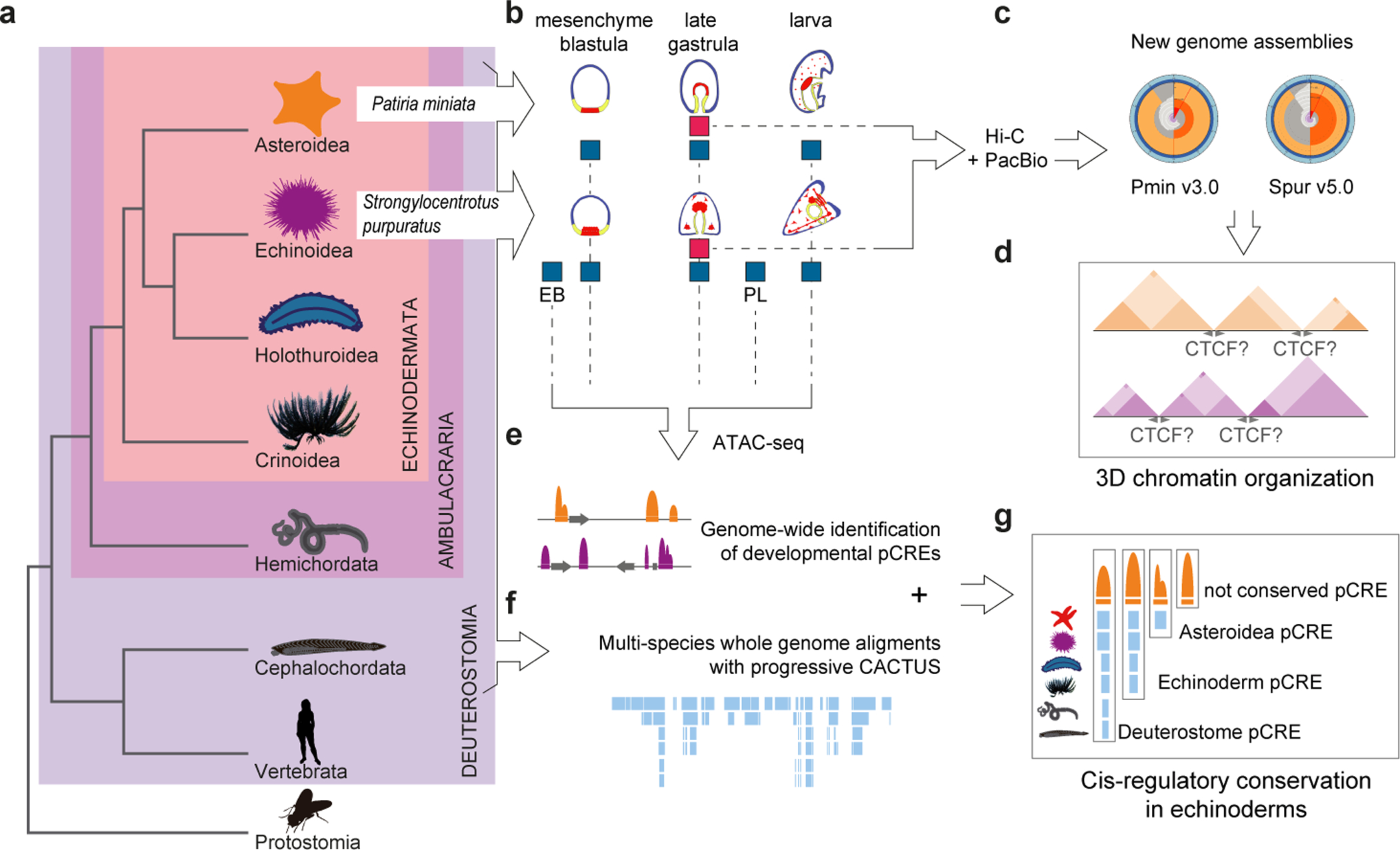
Studying the regulatory genomes of the bat sea star and the purple sea urchin **a,** Schematic tree of animals showing the phylogenetic position of the bat sea star (*P. miniata*) and purple sea urchin (*S. purpuratus*). **b,** Schematic drawings showing the equivalent developmental stages chosen for the generation of ATAC-seq (all stages, dark blue boxes) and Hi-C (late gastrula stage, magenta boxes) data in the bat sea star (*P. miniata*) and purple sea urchin (*S. purpuratus*). EB (early blastula) and PL (prism larva) indicate the two additional sea urchin stages sampled for ATAC-seq. Embryonic germ layers and their derivatives are indicated in blue (ectoderm), red (mesoderm) and yellow (endoderm). **c,** By combining HiC and PacBio data, we generated new genome assemblies of the bat sea star and purple sea urchin (GCA_015706575.1 and GCA_000002235.4, depicted by small snailplots, see full versions in EDF1), allowing us to study 3D chromatin organization in these species (**d**). **e,** Using ATAC-seq, we identified pCREs genome-wide and generated whole genome alignments with multiple outgroup species (**f**), to study the different strata of cis-regulatory conservation in echinoderms (**g**).

To investigate gene regulatory evolution in echinoderms and comprehensively profile CREs together with 3D chromatin organization, we first generated new reference genome assemblies for two of the best developmentally characterized echinoderm species, the bat sea star, *Patiria miniata* ( Asteroidea), and the purple sea urchin, *Strongylocentrotus purpuratus* (Echinoidea) (Fig. 1). We sequenced the samples using PacBio Sequel, achieving 123x coverage for *S. purpuratus*, and 125x for *P. miniata* and generated HiC data for scaffolding. These new *P. miniata* v3.0 (GCA_015706575.1) and *S. purpuratus* v5.0 (GCA_000002235.4) genome assemblies have a total size of 608 Mb and 921 Mb respectively (Fig. 1b) and 96% and 96.3% BUSCO completeness (Extended Data Fig. 1a). The scaffold N50 was 23 Mb for *P. miniata* and 37 Mb for *S. purpuratus* (Extended Data Fig. 1b-d). In *S. purpuratus*, 89.4% of the assembly is contained within 21 main scaffolds larger than 29 Mb and that correspond to the haploid chromosome number reported in cytogenetic studies for this species (n=21^53^) (Extended Data Fig. 1d,f). In the case of *P. miniata*, we could not identify a clear set of main scaffolds that matched the karyotypic number of its closely related sister species, *P. pectinifera* (n=22^54–56^), suggesting that further work will be necessary to obtain a chromosome-grade assembly in this species (Extended Data Fig. 1e). In sum, these two new assemblies represent a great advancement in comparison with previous ones, especially in terms of contiguity, as exemplified by improved N50 values (from 0.052 Mb to 23.1 Mb in *P. miniata* and from 0.42 Mb to 37.3 Mb in *S. purpuratus*).

The genome annotations, both obtained using the NCBI eukaryotic genome annotation pipeline^57^, contain 33,503 gene models (of which 27,447 are protein coding), with 38,426 mRNA transcripts for *S. purpuratus* and 27,818 gene models (of which 23,044 are protein coding), with 35,403 mRNA transcripts for *P. miniata*.

### Chromatin folding in Echinoderms

**Extended Data Figure 2.**
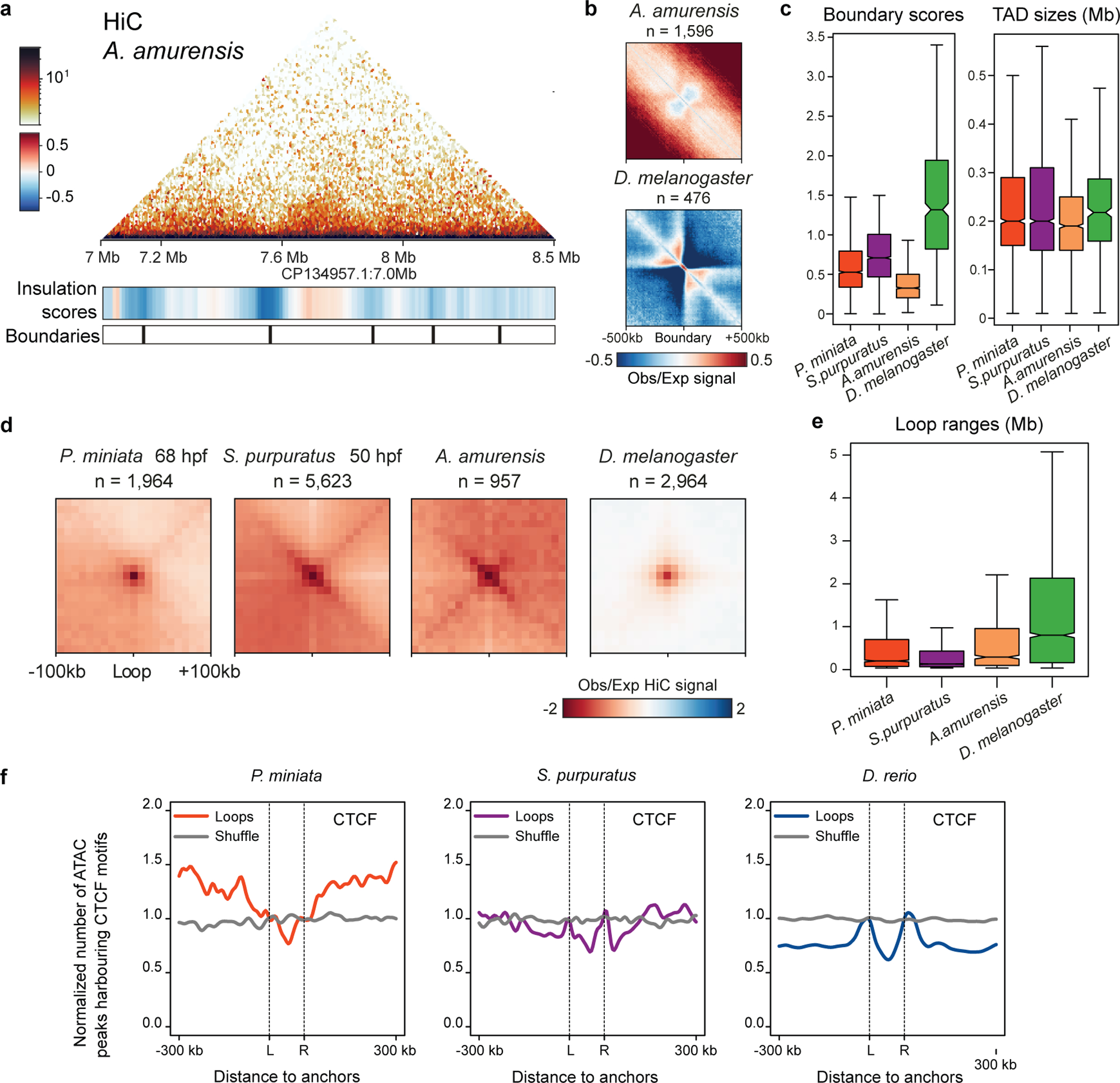
Chromatin domains and loops in echinoderms. **a**, Heatmaps showing normalized HiC signal at 10-kb resolution in *A. amurensis*. Insulation scores and computationally called TAD boundaries are plotted below the heatmaps. **b**, Aggregate analysis of the observed versus expected HiC signal around TAD boundaries called at 10-kb resolution in *A. amurensis* (top) and *D. melanogaster* (bottom). **c**, Boxplots showing the distribution of boundary scores (left) and TAD sizes (right) in *P. miniata* (boundaries n=2,108, TADs n=2042), *S. purpuratus* (boundaries n=3,009, TADs n=2880), *A. amurensis* (boundaries n=1,596, TADs n=1566) and *D. melanogaster* (boundaries n=476, TADs n=484). **d**, Aggregate peak analyses of HiC loops in *P. miniata, S. purpuratus*, *A. amurensis* and *D. melanogaster*. The observed vs. expected HiC signal is shown. **e**, Boxplots showing the distribution of loop ranges in *P. miniata*, *S. purpuratus*, *A. amurensis* and *D. melanogaster*. Number of loops in each species as in d. **f**, Normalized number of ATAC-seq peaks harboring CTCF motifs around HiC loops in *P. miniata* (left), *S. purpuratus* (middle) and *D. rerio* (right). Shuffle control is shown per each graph. Boxplots in c and e show center line, median; box limits, upper and lower quartiles; whiskers, 1.5× interquartile range; notches, 95% confidence interval of the median.

**Extended Data Figure 3.**
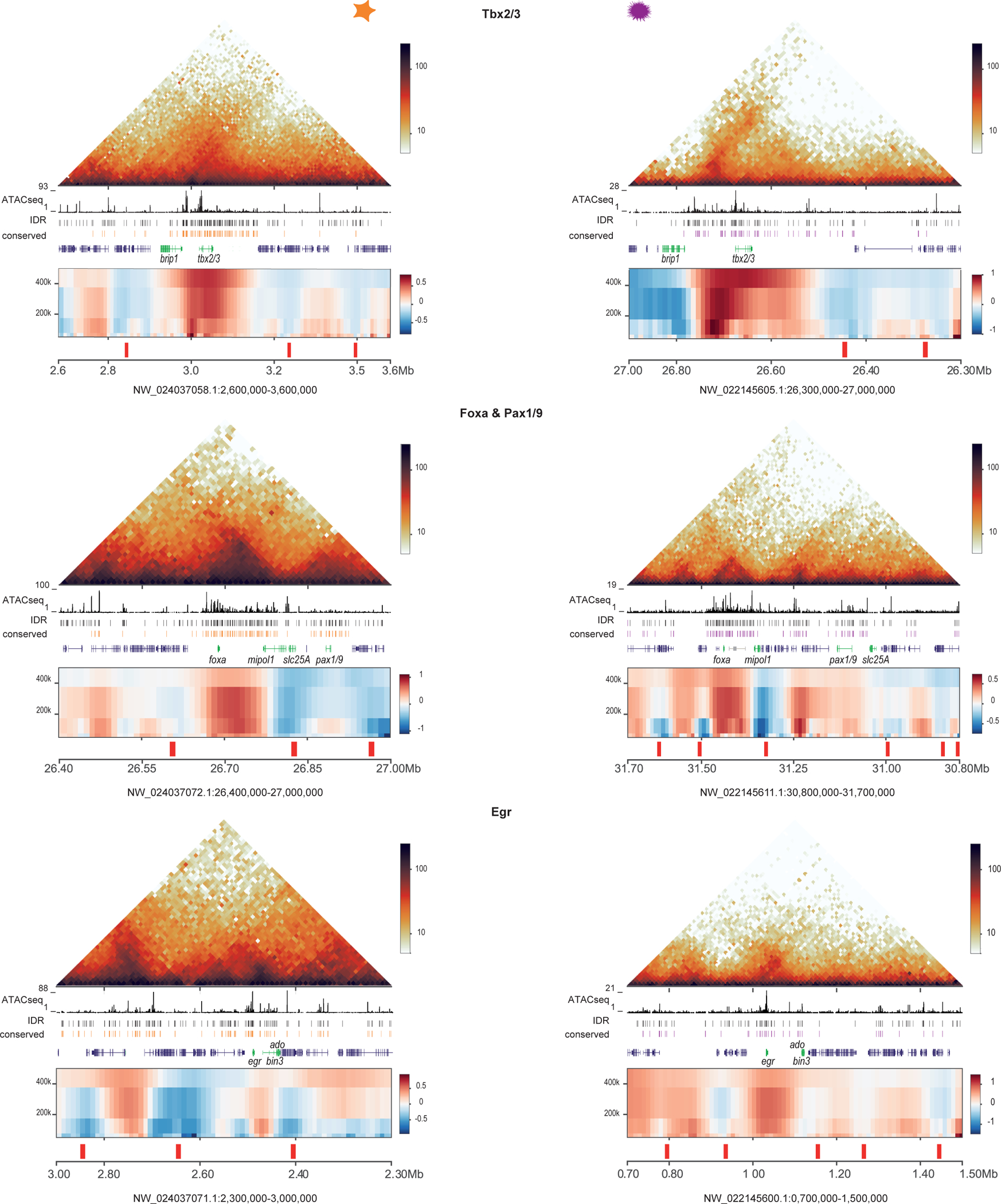
Examples of ancient conserved microsyntenic blocks located within TAD boundaries. Sea star (left panels) and sea urchin (right panels) genomic regions around four developmental genes included within ancient microsyntenic blocks (Tbx2/3, Foxa and Pax1/9, and Egr). From top to bottom: heatmaps showing normalized HiC signal at 10-kb resolution in late gastrula sea star and sea urchin embryos, ATAC-seq signal at late gastrula stage embryos of both species, IDR ATAC-seq peaks (pCREs), conserved ATAC-seq peaks (merging peaks conserved at the Valvatida+Asteroidea strata and Odontophora+Echinoidea strata), gene models (with those included within the conserved syntenic blocks colored in green), insulation scores and computationally called TAD boundaries. Note that the conserved block containing Foxa and Pax1/9 is split in two in the case of sea urchin.

**Extended Data Figure 4.**
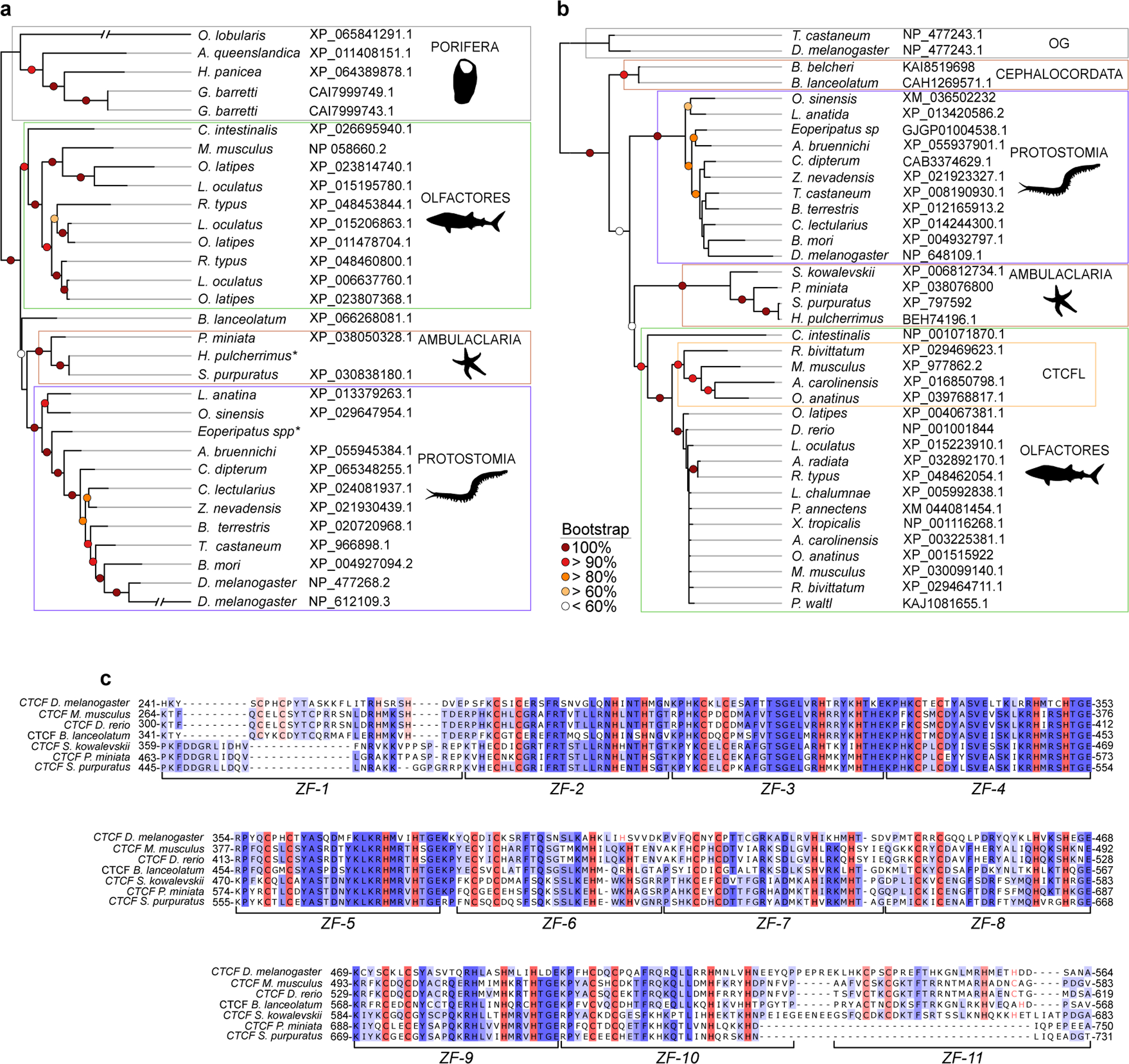
Cohesin and CTCF phylogeny and conservation. **a,** ML phylogenetic tree of cohesin subunit SA1–3 proteins. Sponge SA proteins were used as outgroups. **b,** ML phylogenetic tree of CTCF, including the tetrapod-specific paralog CTCFL (also known as BORIS), the outgroup (OG) branch includes the insect proteins CROOKED LEGS, a zinc finger containing family of transcription factors. **c,** Alignment of CTCF proteins from different bilaterians, showing the region containing its putatively ancestral 11 zinc finger domains, where ambulacrarian species have lost one or two domains.

**Figure 2:**
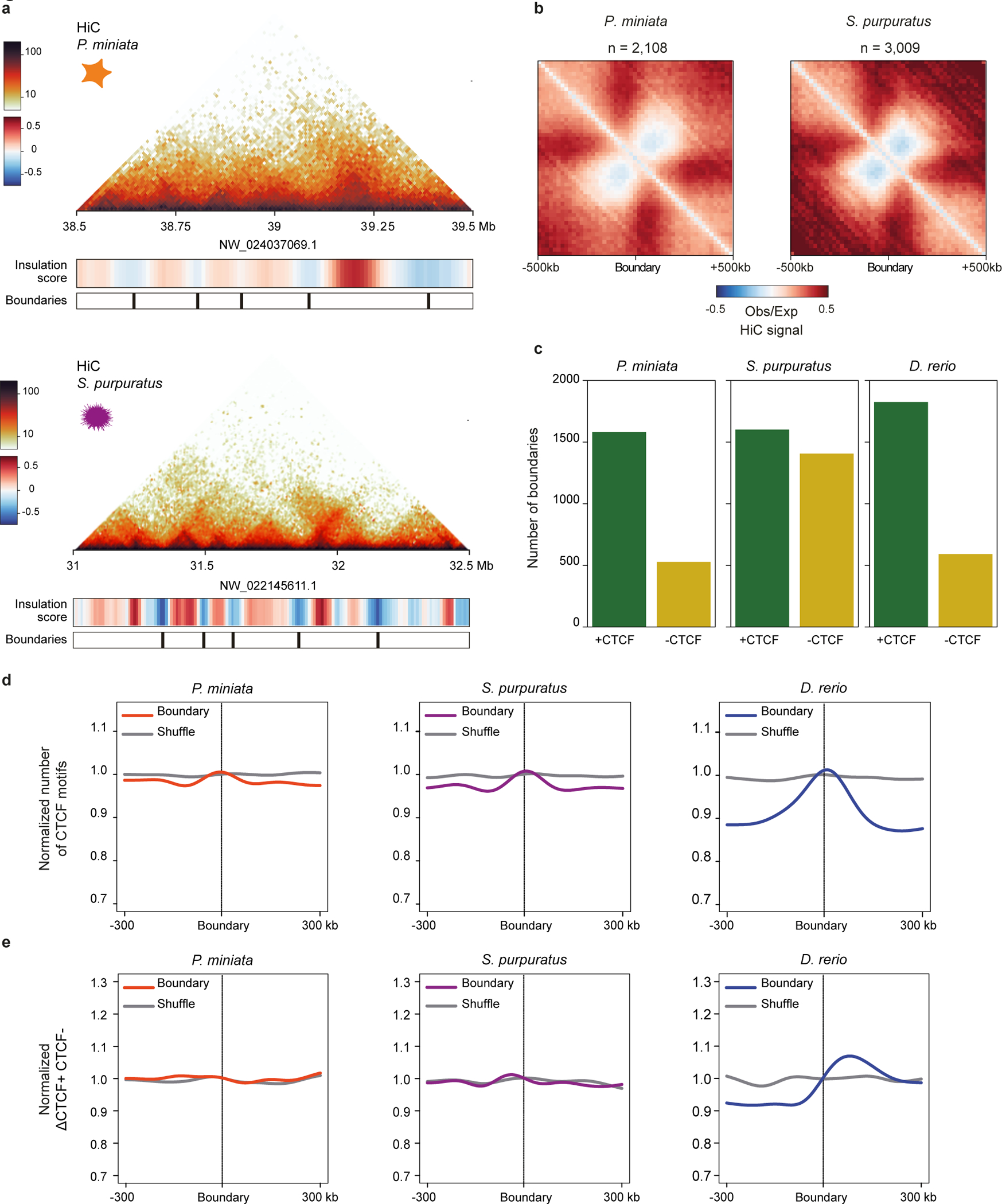
Echinoderm TAD boundaries and CTCF sites **a,** Heatmaps showing normalized HiC signal at 10-kb resolution in *P. miniata* (top) and in *S. purpuratus* late gastrula embryos (bottom). Insulation scores and computationally called TAD boundaries are plotted below the heatmaps. **b**, Aggregate analysis of the observed versus expected HiC signal around TAD boundaries called at 10-kb resolution in *P. miniata* (left) and *S. purpuratus* late gastrula embryos (right). **c**, Number of boundaries harboring or not CTCF motifs. **d**, Average number of CTCF motifs within ±300 kb around boundaries (colored) and shuffled controls (grey). **e**, Normalized difference of CTCF motifs between forward and reverse strands within ±300 kb around boundaries (colored) and shuffled controls (gray).

To characterize chromatin folding in our two echinoderm species, we used HiC data from late gastrula stage of sea star and sea urchin embryos and compared them with data from other animal lineages. Using a 10kb window, we first observed that at the sub-megabase scale the *P. miniata* and *S. purpuratus* genomes fold in self-insulated domains reminiscent of Topologically Associated Domains (TADs) observed in other animals, such as chordates, fruitflies, annelids and cephalopods^22,58–65^ (Fig. 2a,b). We used insulation scores and boundary scores to define a comprehensive set of putative TADs and TAD boundaries, and we also called chromatin loops, identifying 2,108 and 3,009 boundaries and 1,964 and 5,623 loops in *P. miniata* and *S. purpuratus*, respectively. TADs and loops were also present in an additional very distantly related sea star species, the Northern Pacific sea star *Asterias amurensis*, where we found 1,596 TAD boundaries and 957 loops using publicly available HiC data for this species^66^ (Extended Data Fig. 2a-d). The three echinoderm species had more TADs than fruitflies, while their size distributions were relatively similar (ranging from a median of 190 Kb in *A. amurensis* to 200 Kb in *S. purpuratus* and *P. miniata*, and 210 Kb in *D. melanogaster)*. In contrast, chromatin loop ranges were larger in *D. melanogaster* (median of 800 Kb compared to 290 Kb in *A. amurensis*, 200 Kb in *P. miniata* and 130 Kb in *S. purpuratus*) (Extended Data Fig. 2b-e). Importantly, previously identified conserved microsyntenic pairs, which are usually associated with developmental gene families, such as Pax1/9, Foxa, Tbx2/3, and Egr^13,16^, that also show a high abundance of conserved non-coding sequences^3^, were included within *P. miniata* and *S. purpuratus* TADs, supporting that these domains could have a role in long-range regulation in echinoderms (Extended Data Fig. 3). We then investigated which molecular mechanisms could be responsible for boundary formation in echinoderms. First, we confirmed that both *P. miniata* and *S. purpuratus* genomes have orthologs of the two main proteins involved in loop extrusion and boundaries in vertebrates, CTCF and cohesin subunit SA1–3 (Extended Data Fig. 4a,b). CTCF orthologs in echinoderms and their sister lineage, hemichordates, were more divergent than those of other bilaterian phyla, and were characterized by the loss of one (in hemichordates) or two (in echinoderms) of the eleven Zn-finger domains ancestrally present in bilaterian CTCF proteins (Extended Data Fig. 4c), as noted previously in another sea urchin species, *Hemicentrotus pulcherrimus*^67^. Next, we checked if CTCF could function similarly to vertebrates in echinoderm TAD boundaries, by looking for CTCF motif enrichment in *P. miniata*, *S. purpuratus* and the zebrafish *Danio rerio* as a positive control. Both sea star and sea urchin showed a CTCF motif enrichment in boundaries, although much lower than in the vertebrate, especially in *S. purpuratus* where CTCFs motifs were absent in nearly half of the boundaries (Fig. 2c,d). We next assessed if these CTCF motifs in echinoderm TAD boundaries displayed the typical vertebrate pattern with divergently oriented CTCF binding sites at each side of the boundaries^19–22^. In contrast with zebrafish, the orientation of sea star and sea urchin CTCF motifs at boundaries did not show any orientation pattern, with distributions indistinguishable from the shuffled controls (Fig. 2e). Furthermore, there was no enrichment of CTCF motifs at sea star or sea urchin loop anchors, suggesting that CTCF functions differently in these species than in vertebrates (Extended Data Fig 2f).

### *The Developmental Regulatory Genome of* P. miniata *and* S. purpuratus

**Extended Data Fig. 5.**
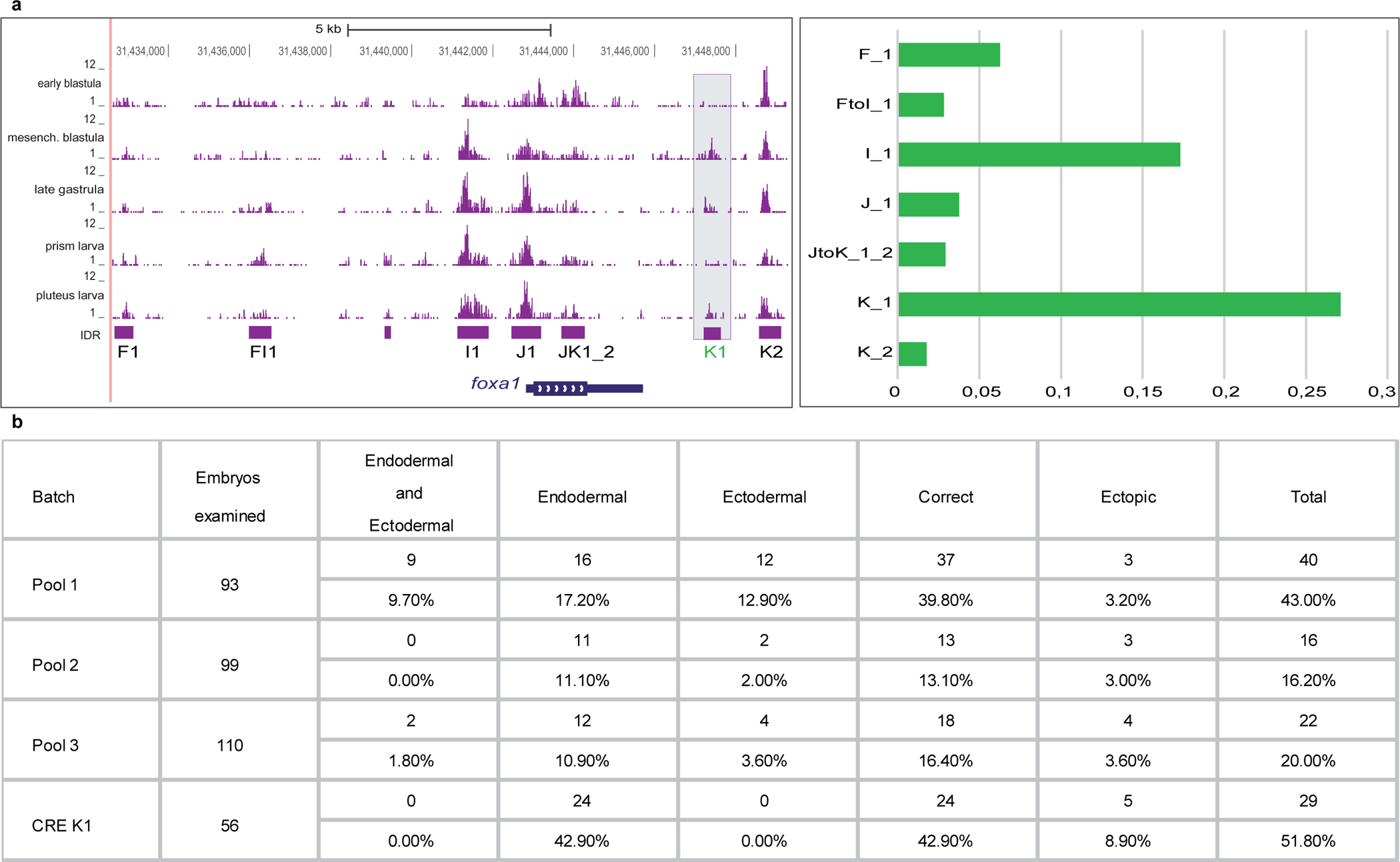
Functional assays of *foxa1* pCREs. **a,** Screenshot of sea urchin UCSC browser with ATAC-seq tracks around the *foxa1* (LOC110977664) gene and pCREs. Scoring table for embryos injected with *foxa1* CRE-Tag constructs at mesenchyme blastula stage. **b,** Relative GFP expression levels driven by *foxa1* CREs in sea urchin embryos at mesenchyme blastula stage. The embryos injected with the pool of *foxa1* CREs showing GFP expression in various domains were scored, highlighting the distribution of embryos with expression in both ectoderm and endoderm, only ectoderm or only endoderm, as well as the ones exhibiting GFP expression in regions ectopic to *foxa1* expression. This was also concordant with scoring done for the FIJ concatenate^69^ since the majority of the expression is in the endoderm with oral ectoderm expression exhibited by fewer embryos.

**Fig. 3:**
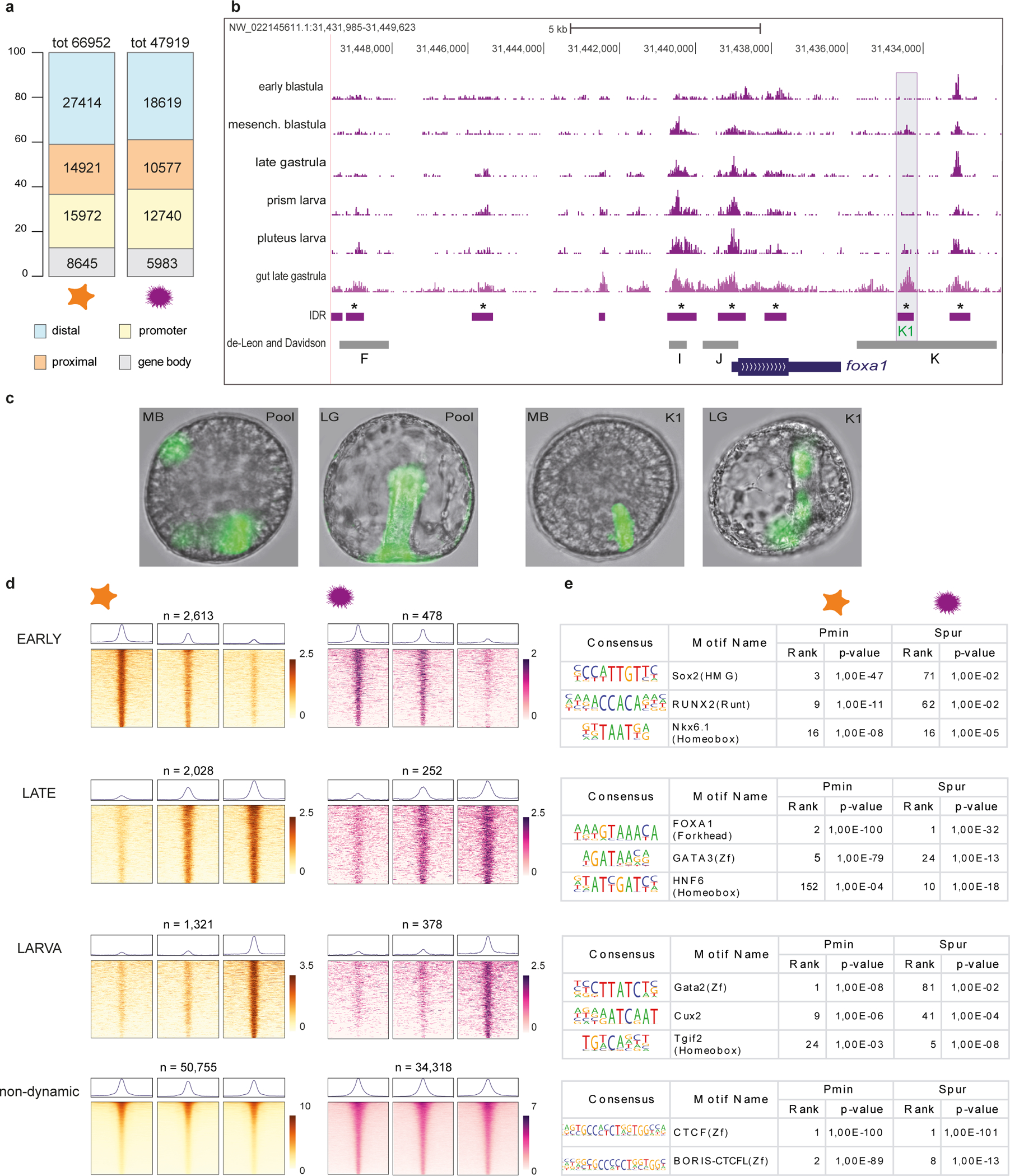
Regulatory annotation of echinoderm genomes using chromatin accessibility **a,** Numbers and proportions of open chromatin regions of both species according to their genomic location: promoters, for regions overlapping a gene TSS; proximal, within 5-kbp upstream of (but not overlapping with) a TSS; distal, not in the aforementioned categories. **b,** Screenshot of sea urchin UCSC genome browser with ATAC-seq tracks showing the correspondence between published^69^ *foxa1* (LOC110977664) CREs (gray bars) and ATAC-seq peaks (pCREs, purple bars). Asterisks indicate the seven pCREs tested in (c). **c,** From left to right, GFP expression driven by the *foxa1* pCREs pool (panels first and second) and the single K1 pCRE (third and fourth) at mesenchyme blastula (MB) and late gastrula (LG) stages. **d,** Heatmaps of clusters of pCREs of sea urchin (left panels, in purple) and sea star (right panels, in orange) that are more accessible at early (top panels), late (second row panels) and larva (pluteus/bipinnaria, third row panels) stages, as well as clusters of non-dynamic pCREs that did not show changes in accessibility across the sampled stages (bottom panels). Each heatmap column shows normalized ATAC-seq nucleosome-free signal for each stage ordered by developmental time (left: mesenchyme blastula, middle: late gastrula, right: bipinnaria/pluteus larva) over a 2 kb window centered in the midpoint of each of the pCREs, each heatmap horizontal line corresponds to a single pCRE. Average ATAC-seq signal profiles of all the pCREs in each stage are shown on top and the number of pCREs contained in each cluster are indicated at the lower right corner of each panel. **e**, Examples of TFBMs enriched in each of the temporal pCREs clusters that occupy top-ranked positions in both or just one of the two echinoderm species. The families of their corresponding TFs are indicated in brackets.

We next annotated the developmental regulatory genome of the two echinoderm species, by identifying putative CREs (pCREs) through profiling of open chromatin regions with ATAC-seq (Assay for Transposase-Accessible Chromatin using sequencing). ATAC-seq peaks are widely used as CRE proxies across diverse animal lineages^7,12,45,52,68^. We performed ATAC-seq experiments in sea urchin and sea star whole-embryos at different timepoints ranging from blastula to larva, including three equivalent developmental stages between the two species (Fig. 1b), and identified a total of 66,952 and 47,919 pCREs in *P. miniata* and *S. purpuratus*, respectively (Fig. 3a, Supplementary Table 1). The genomic distribution of these elements was relatively similar between the two species, with the majority of pCREs (~40%) laying in distal locations with respect to gene promoters or gene bodies (Fig. 3a). Sea urchin pCREs consistently recapitulated previously described CREs in this species, with 44% (18 out of 41) of previously tested elements overlapping with pCREs (Supplementary Table 2). A paradigmatic example was the *S. purpuratus foxa1* gene, where we were able to identify pCREs that overlapped all four previously described regulatory elements in the intergenic regions surrounding it (Modules *F*, *I*, *J* and Region *K*, indicated by grey boxes in Fig. 3b)^69^, but with higher resolution, as some of the previously identified modules could actually be divided into several pCREs (Region *K*) or their length were longer or shorter than the accessible regions (Modules *F*, *I*, *J*). We also found three additional elements that had not been reported before, two pCREs located between modules *F* and *I* and another one between *J* and *K* (Fig. 3b). We tested seven of these *foxa1* pCREs in transient transgenic reporter assays^70^ in sea urchin embryos (indicated by asterisks in Fig. 3b). They drove GFP expression recapitulating the endogenous *foxa1* expression, both injected as pools, containing all seven pCRE-GFP constructs together, or individually (Fig. 3c). In the mesenchyme blastula, the construct pool drove GFP expression in the endoderm and oral ectoderm, with less than 3% of the injected embryos showing ectopic expression (mostly in the primary mesenchyme cells and misdeveloped embryos) (Extended Data Fig. 5a,c). In late gastrula, the expression was visible in the whole gut, equivalent to the expression patterns driven by previous construct concatenates (the FIJ CRE concatenate as described in^69^ (Fig. 3c). Furthermore, quantitative expression assessment of the pCREs injected individually, showed that the sub-region K1 of the Region *K*, which was not previously analyzed in detail, was the strongest *foxa1* element tested, and its activity alone was enough to recapitulate all *foxa1* endodermal expression, consistent with the high accessibility of K1 in previously published ATAC-seq data from isolated gut samples (Extended Data Fig. 5b)^51^. These results support that pCREs act as developmentally regulated transcriptional enhancers and are a good proxy for bona-fide CREs^7^.

To better understand modules of regulation during development, we looked for developmentally dynamic pCREs, that is, open chromatin regions that change their accessibility during development. We found that 24% (16,197) and 4% (1,513) of *P. miniata* and *S. purpuratus* pCREs were differentially accessible in at least one of the three developmental stages. The remaining “non-dynamic” pCREs presumably contained constitutive pCREs as well as pCREs that change their accessibility at other stages not sampled in our study. We then clustered these developmentally dynamic pCREs according to their signal profiles across the three matched developmental stages of the two species. In both echinoderms, we identified three clusters of dynamic pCREs whose accessibility changes recapitulated the temporal progression across the three stages: “early”, “late” and “larva” clusters (Fig. 3d). In *P. miniata* and *S. purpuratus* “early” clusters corresponded to 16% and 31% of all dynamic pCREs, respectively, “late” clusters to 13% and 17% and “larva” clusters to 8% and 25% (Fig. 3d). To characterize the trans-regulation connected to these clusters of dynamic pCREs as well as the corresponding sets of “non-dynamic” pCREs, we performed transcription factor binding motif (TFBM) enrichment analysis using a set of vertebrate TFBMs. These results showed clear similarities among the top-ranked TFBMs in the two species in some of the clusters (Fig. 3e, Supplementary Table 3). For instance, the motifs of Foxa genes were highly enriched in the late clusters of both species, highlighting the crucial role of this gene family in endoderm formation during gastrulation^12,69,71–73^, while CTCF, together with its vertebrate-specific paralog, BORIS/CTCFL^74,75^, was the top-ranked motif of the non-dynamic clusters, consistent with a putative role of CTCF as an architectural protein, as suggested by its enrichment at TAD boundaries (Fig. 3e). However, differences in the temporal usage of TFBMs were much more prevalent, and the majority of top motifs in one species occupied much lower ranks in the other. This was the case of Sox motifs in early pCREs, which ranked much higher in sea star than in sea urchin, the motifs of several families of homeobox factors, such as Pitx and Otx, with more prominent positions in the early cluster of sea urchin, and Gata motifs, which were highly enriched in larva pCREs only in sea star (Fig. 3e, Supplementary Table 3).

### Regulatory conservation in echinoderms and deuterostomes

**Extended Data Fig. 6.**
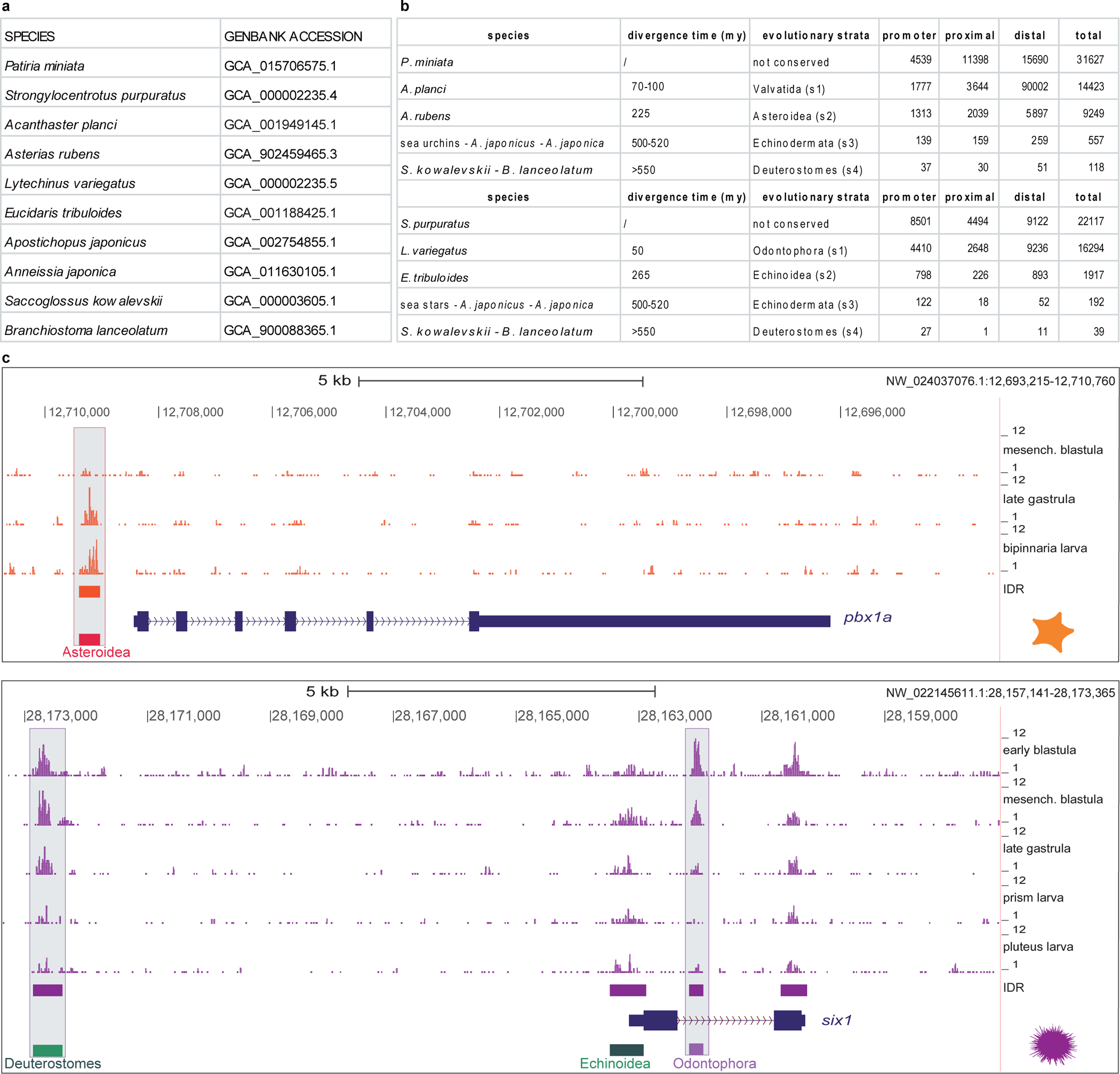
Evolutionary strata and developmental dynamics of sea urchin and sea star pCREs. **a,** Accession numbers of the genome assemblies included in the alignments. **b,** Counts of pCREs according to their evolutionary strata. **c,** Genome browser screenshots around the *P. miniata pbx1a* (LOC119720117, top panel) and the *S. purpuratus six1* (LOC110974175, lower panel) genes showing ATAC-seq tracks from different developmental stages (in orange and purple, respectively), IDR peaks (orange and purple bars) and gene models (dark blue). A conserved Asteroidea pCRE (red bar) with late developmental dynamics and Deuterostome and Odontophora pCREs (green bars) with early developmental dynamics are highlighted with gray boxes.

**Figure 4.**
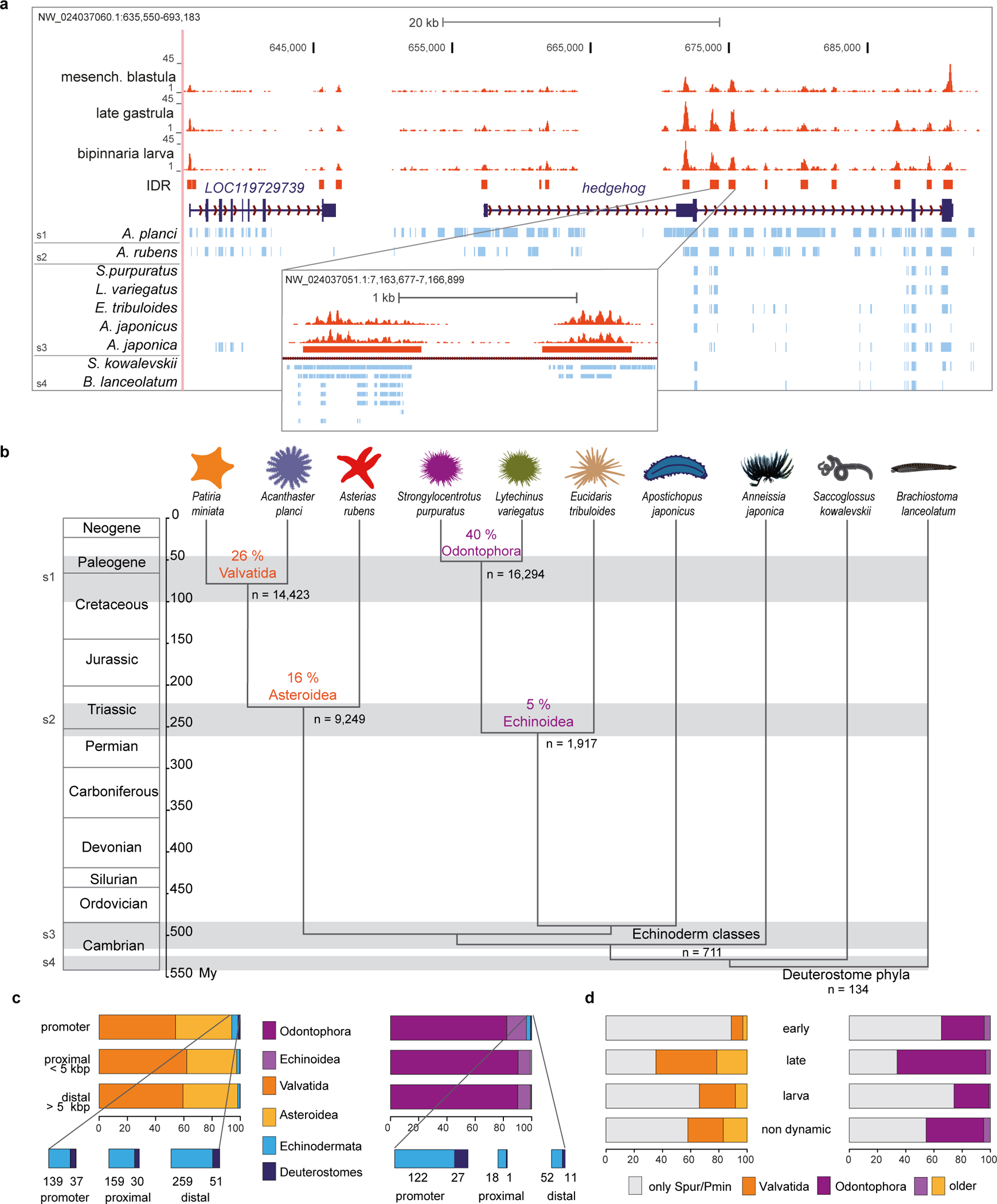
Evolutionary conservation of pCREs in echinoderms **a**, Genome browser screenshot around the *P. miniata hh* gene (LOC119729696) showing ATAC-seq tracks from different developmental stages (orange) and the blocks of conserved sequences with other species included in the multiple genome alignment (light blue). The four evolutionary strata are indicated as “s1” to “s4”. Gene models are shown in dark blue. **b,** Schematic phylogenetic tree of the deuterostome species included in the genome alignments showing the percentage and total number of sea star (orange) and sea urchin (purple) conserved pCREs at each evolutionary strata (indicated by gray bars, s1 to s4). **c,** Genomic distribution of pCREs according to their evolutionary strata. Distributions are shown in orange for sea star and in purple for sea urchin. **d**, Proportions of conserved elements in each of the sea star and sea urchin pCREs temporal clusters (early, late, larva and non-dynamic).

To study the evolution of sea urchin and sea star pCREs, we generated a multi-species alignment of their genomes together with additional genome sequences from multiple echinoderm and outgroup species. We selected species spanning a wide range of phylogenetic distances and divergence times from our target species, to be able to differentiate between conserved pCREs of relatively recent origin with a similar age to those previously identified in flies and nematodes, from deeply conserved pCREs such as those present in vertebrates, as well as transphyletic pCREs conserved across different phyla (Fig. 4, Extended Data Fig. 6a,b, Supplementary Tables 4,5). This allowed us to detect blocks of conserved sequences between the genomes of the different species and, therefore, identify which sea urchin and sea star pCREs lay within these conserved blocks and determine their level of conservation. With this, we recognized four evolutionary strata (s) among the conserved regions. First, s1, the most recent ones, shared with other species of the same order/superfamily than our reference species (*Acanthaster planci* from Valvatida for *P. miniata* and *Lytechinus variegatus* from Odontophora for *S. purpuratus*) and dating back at least to ∼50–80 mya^76^. Second, s2, conserved with the most distantly related species within each of their respective echinoderm classes, Asteroidea (with *Asterias rubens*) and Echinoidea (with *Eucidaris tribuloides*), that separated at least ∼200–265 mya^77,78^. Third, s3, conserved across different echinoderm classes, including the two classes of our species as well as others such as Holothuroidea (*Apostichopus japonicus*) and Crinoidea (*Anneissia japonica*), with divergence times of approximately 520 mya^79^. And fourth, s4, conserved with other deuterostome phyla, such as hemichordates (*Saccoglossus kowalevskii*) and chordates (*Branchiostoma lancelolatum*), which separated from echinoderms more than 550 mya^80,81^. With this classification scheme, we assigned all sea star and sea urchin pCREs to a particular age class. For example, in the second intron of the sea star *hedgehog* gene (Fig. 4a), we found an Echinodermata pCRE, conserved across different echinoderm classes, plus another restricted to asteroidean species (i.e., an Asteroidea pCRE). About 55% of sea urchin and sea star pCREs were not conserved with the other species, indicating a high degree of sequence turnover, as it has been previously reported in other animal lineages^29,82,83^. Furthermore, among conserved pCREs (Fig. 4b and Extended Data Fig. 6b), the majority of them appear to have a relatively recent origin (~50–80 mya), and are only conserved within Valvatida (26%) and Odontophora (40%), with a minimum age of a similar magnitude than the most ancient conserved CREs identified in nematodes and fruit flies, where only recent evolutionary elements are present^31,33,34^. Remarkably, in stark contrast to these two ecdysozoan lineages, we also found that 16% (9,249 pCREs) and 5% (1,917 pCREs) of sea star and sea urchin pCREs were very ancient elements older than 200–265 my, predating the origin of the Echinoidea and Asteroidea crown groups in the late Permian and the Triassic (Fig. 4b). To date, this type of regulatory conservation, with several thousand elements that are at least several hundred my old, has only been reported in the vertebrate lineage^40–44^. Finally, we also found a small number of pCREs with extremely deep conservation, shared by different classes of echinoderms (711 elements) or across other deuterostome phyla (134 elements, 89 with hemichordates and 45 with chordates) and whose origin dated back at least to the Cambrian, more than 500 mya^84^. We next checked the genomic distribution of the different evolutionary strata of conserved pCREs. The most recent Valvatida and Odontophora elements were almost equally distributed across the different types of genomic locations, with a slightly higher prevalence in distal and proximal positions versus promoter locations (Fig. 4c, Extended Data Fig. 6b). The older class-specific Asteroidea and Echinoidea elements were also similarly prevalent in different genomic locations, although in contrast to the previous age category, they were more frequently found at promoter positions. On the other hand, the most ancient pCREs, conserved between echinoderm classes or deuterostome phyla, were mostly overlapping promoter regions in *S. purpuratus* and distal regions in *P. miniata*. We then studied the degree of conservation of the temporal dynamic pCREs we identified in the previous section, as well as the non-dynamic, potentially constitutive, pCREs (Fig. 4d, Extended Data Fig. 6c). Interestingly, the conservation of developmental dynamic regions indicated that “early” and “larva” pCREs are the least conserved, while “late” pCREs were the most conserved in both species (Fig. 4d). “Non-dynamic” pCREs showed an intermediate pattern, with a lower percentage of conserved elements than “late” clusters but higher than “larva” and “early” clusters.

**Extended Data Fig. 7.**
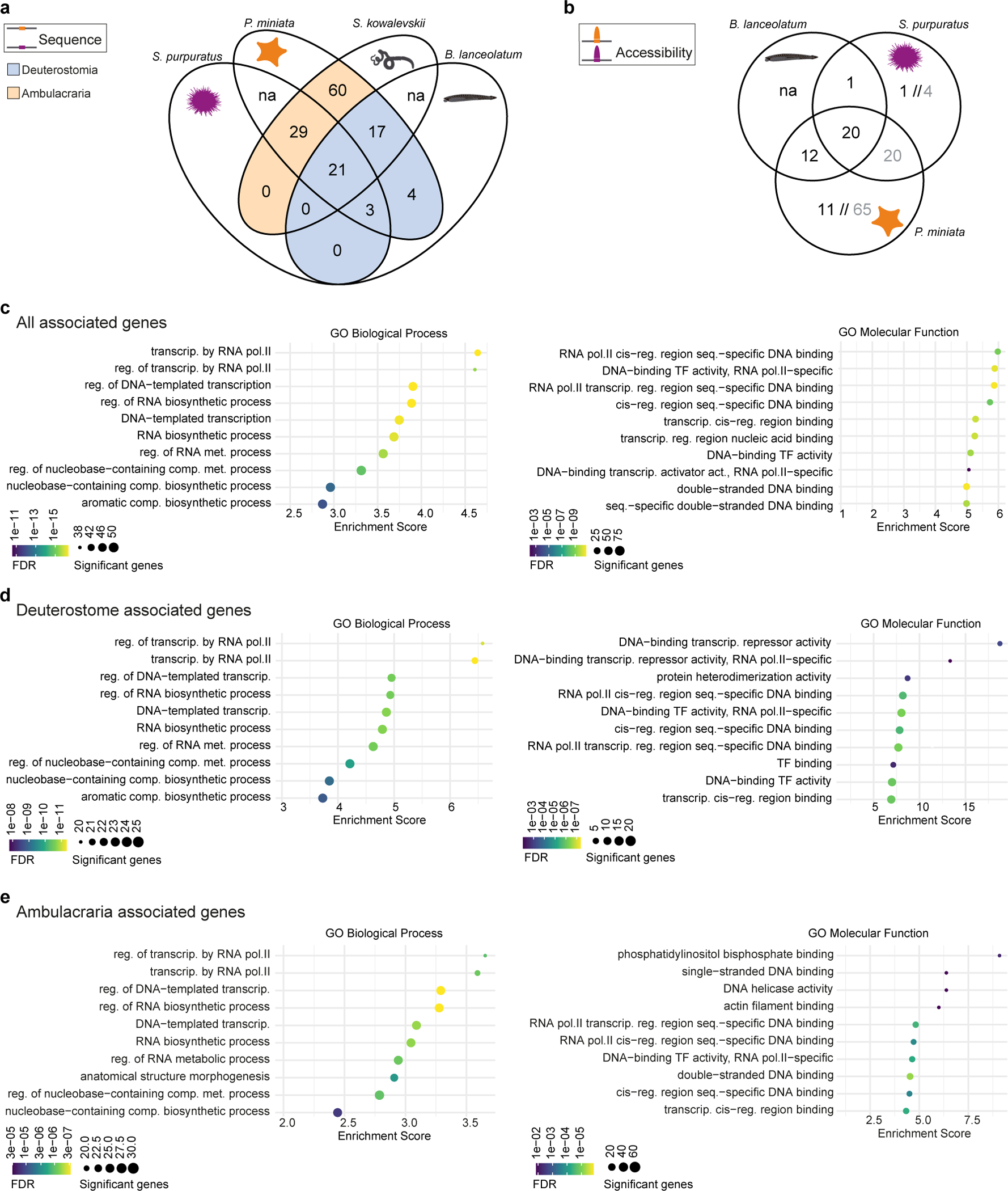
Sequence and accessibility conservation of transphyletic pCREs and GO term enrichments of their associated genes. **a,** Venn diagram with the number of conserved transphyletic pCREs present in each species. pCREs ancestral to deuterostomes and to ambulacrarians are shadowed in blue and orange, respectively. n.a. (not applicable) labels pCRE sharing between species pairs not addressed in this plot: those between amphioxus and hemichordates, which were not directly investigated in the present work, and pCREs shared between sea star and sea urchin, where shared conserved pCREs from the echinoderm stratum were not included to avoid confusions with transphyletic pCREs. **b,** Venn diagram with the number of transphyletic pCREs showing shared chromatin accessibility in sea star, sea urchin and amphioxus embryos. Deuterostome and ambulacrarian pCREs are indicated in black and gray, respectively. **c, d, e,** Top ten significantly enriched GO terms of genes associated with all transphyletic pCREs (c), and with the subsets of pCREs ancestral to deuterostomes (d) and ambulacrarians. Biological Process and Molecular Function ontologies are indicated in the left and right panels, respectively.

**Extended Data Fig. 8.**
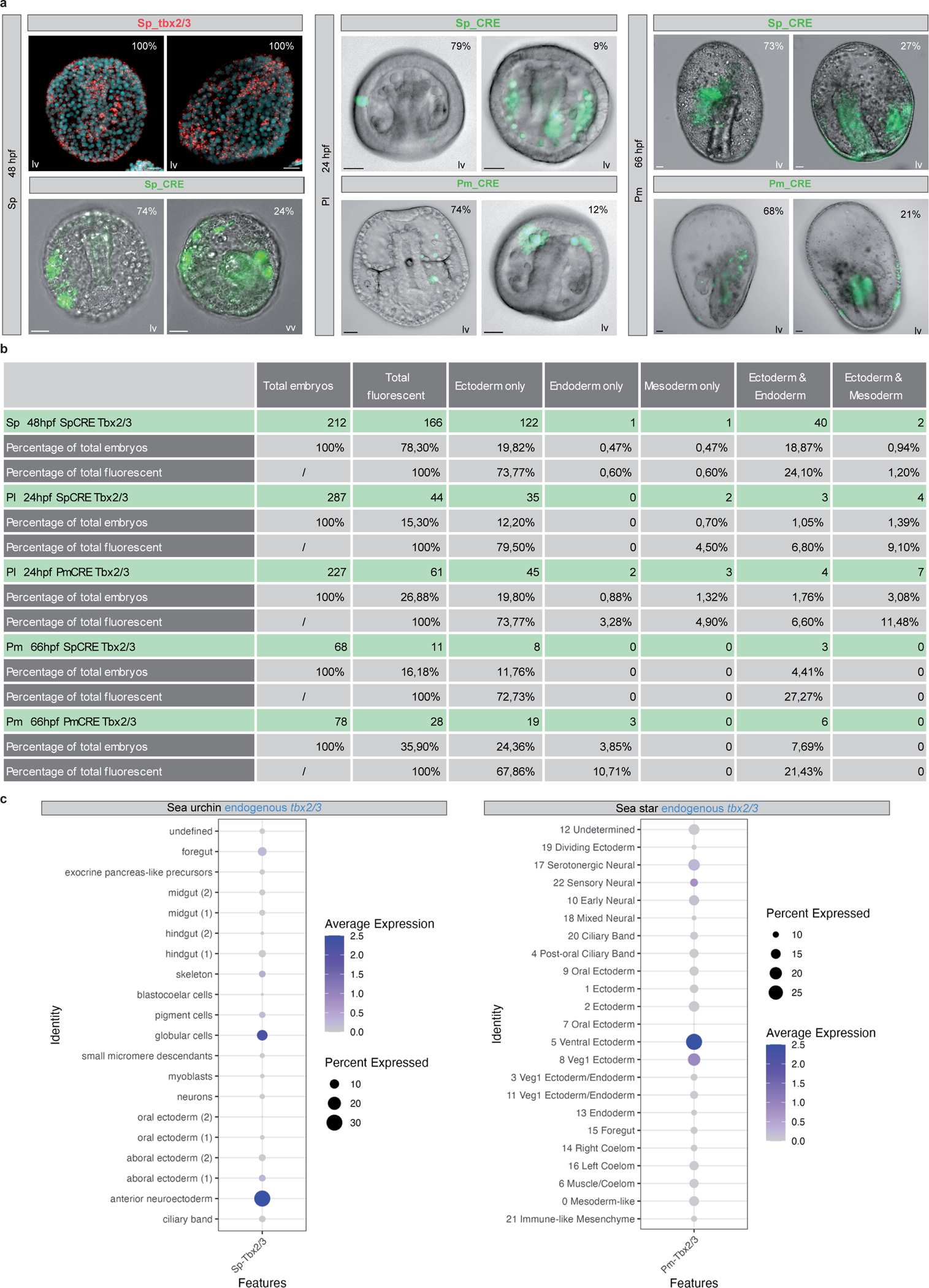
Functional assays of Tbx2/3 deeply conserved CRE. **a,** Numbers of embryos examined in functional assays with the two different CREs and percentages of expression in *S. purpuratus* (Sp), *P. lividus* (Pl), *P. miniata* (Pm). Microinjection experiments were performed using at least two independent batches of embryos and for each experiment at least 30 embryos were analyzed. **b,** Expression pattern of *tbx2/3* by fluorescent in situ hybridization in *S. purpuratus* late gastrula (top left, focus on the ectoderm) and functional assays of *S. purpuratus* and *P. miniata* CREs in different species (*S. purpuratus* (Sp), *P. lividus* (Pl), *P. miniata* (Pm)) by GFP fluorescence. Scale bar 20 µm; lv, lateral view; vv, ventral view. **c**, The dot plots highlight the expression of endogenous *tbx2/3* as detected from scRNA-seq data analysis of gastrula stages in *S. purpuratus* ^51^, left column, and from snRNA-seq data analysis of gastrula stages in *P. miniata* ^91^, right column.

**Figure 5.**
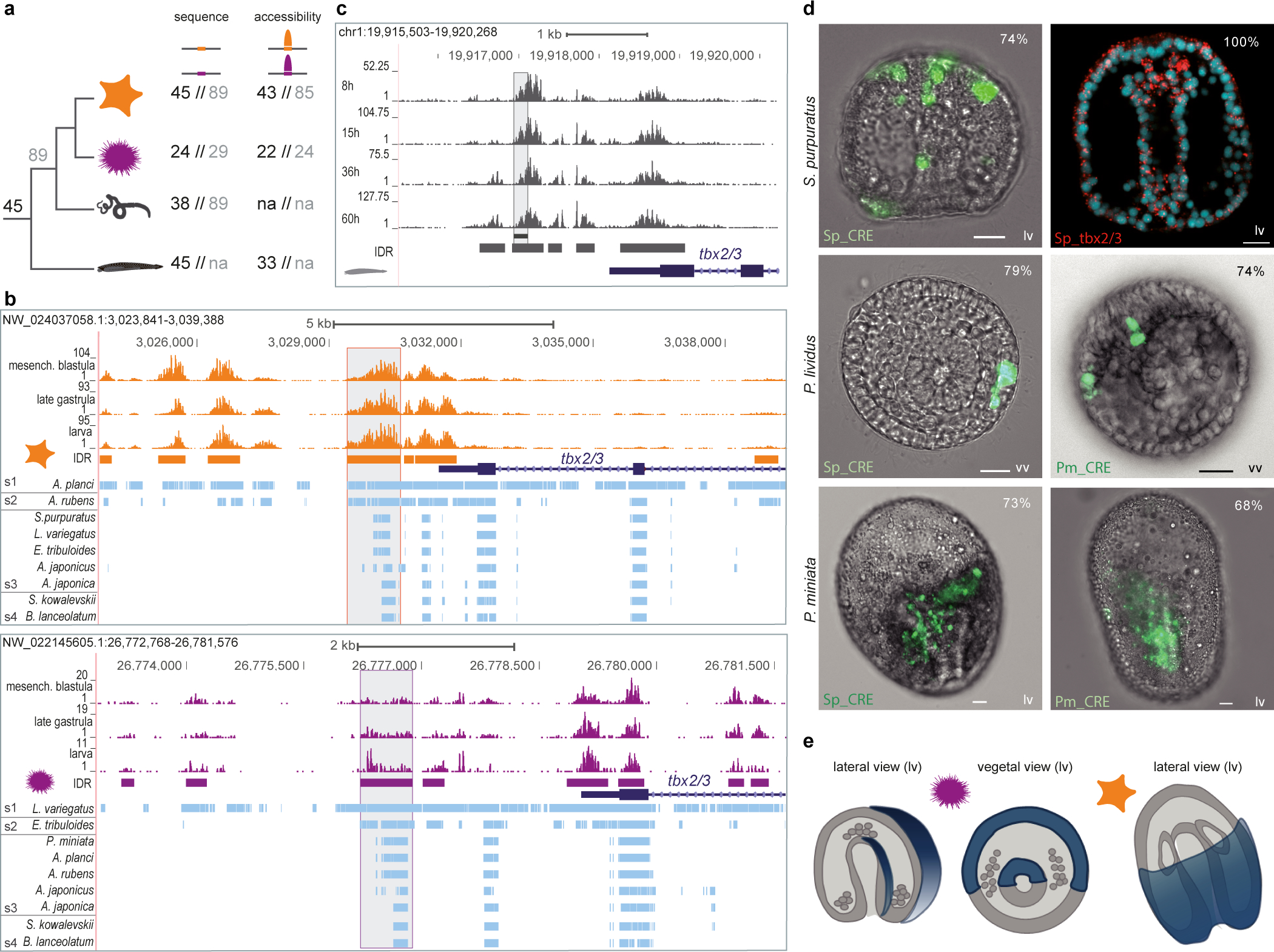
Deeply Conserved pCREs in deuterostomes. **a,** Numbers of transphyletic pCREs across deuterostome (numbers in black) and ambulacrarian (numbers in gray) phyla with conserved sequence and chromatin accessibility in sea star, sea urchin, hemichordate and the chordate amphioxus. n.a.: not applicable. **b,** Genome browser screenshots around the proximal promoter region of the Tbx2/3 genes of sea star and sea urchin. ATAC-seq tracks are colored in orange (sea star) and purple (sea urchin), gene models are shown in dark blue, and blocks of conserved sequences in the multiple genome alignment are indicated in light blue. In both screenshots the deeply conserved CRE tested in functional assays is highlighted by a light gray box. **c,** Genome browser screenshot around the proximal promoter region of the amphioxus *Tbx2/3* gene. ATAC-seq tracks are colored in gray and IDR ATAC-seq peaks are indicated by gray bars. The Tbx2/3 conserved sequence and the gene models are indicated as in (b). Note that this screenshot corresponds to a newer version of amphioxus genome assembly (BrLan3 PRJEB49647)^148^, as this region was split between two scaffolds in the version used for the conservation analyses. **d,** GFP expression driven by the *S. purpuratus tbx2/3* CRE in *S. purpuratus* (top left panel), *P. lividus* (central top panel) and *P. miniata* (right top panel) late gastrulae, and by the *P. miniata tbx2/3* CRE in *P. lividus* (central bottom panel) and *P. miniata* (right bottom panel) late gastrulae. Percentages represent the number of embryos showing expression in a specific domain compared to the total number of fluorescent embryos detected in the assay. Microinjection experiments were performed using at least two independent batches of embryos and for each experiment at least 30 embryos were analyzed (for details see table in Extended Data Fig. 8). Scale bar 20 µm. The GFP expression patterns are consistent with the endogenous expression of *tbx2/3* in the three species, as shown in the fluorescent *in situ* hybridization of *tbx2/3* in *S. purpuratus* late gastrula (top right panel) and as deduced from current (*S. purpuratus*) and previously published *in situ* hybridization data (*P. lividus*, ^149^) and analysis of scRNA-seq data (*P. miniata*, Extended Data Fig. 8^91^). **e,** Schematic drawings representing shared domains between sea urchin and sea star Tbx2/3 expression (in blue) in wild type and transgenic embryos.

Sequence conservation might not necessarily imply functional conservation. Therefore, we also checked whether the sea star and sea urchin deeply conserved transphyletic elements also had conserved chromatin accessibility in these two echinoderms as well as in one of the non-echinoderm phyla included in our analyses, the amphioxus *Branchiostoma lanceolatum*, an invertebrate chordate with previously available ATAC-seq data^7^. Almost all of the 45 elements conserved at the sequence level between echinoderms and chordates also had conserved chromatin accessibility in each of these three species, with 100% (45 out of 45) of conserved accessibility in sea star, 92% (22 out of 24) in sea urchin and 73% (33 out of 45) in amphioxus. We found a similar pattern in the rest of sequence-conserved transphyletic elements, the 89 pCREs conserved with the other non-echinoderm phyla we investigated, the hemichordate *S. kowalevskii*, with 100% and 83% of them showing conserved accessibility in sea star and sea urchin, respectively (Fig. 5a, Supplementary table 6). The few cases where we could not detect conserved accessibility (25 pCREs, less than 10% of the total set of 232 regions present in sea star, sea urchin and amphioxus) could be due to pCREs that are active at different developmental times to the ones sampled by ATAC-seq in these three species or the fact that the cell populations where these pCREs are active are too small to be reliably identified as IDR ATAC-seq peaks using whole embryo datasets. Alternatively, they could also represent genuine cases of functional divergence, indicating that sequence conservation might not necessarily imply functional conservation. In any case, our results showed that the overwhelming majority of sequence-conserved transphyletic pCREs also had conserved chromatin accessibility, suggesting that the loss of sequence conservation is more prevalent than the loss of conserved accessibility alone, as in the case of the sea urchin, where we could only detect 53 of the 134 ancestral pCREs conserved between the sea star and the non-echinoderm phyla (Fig. 5a, Extended Data Fig. 7a,b Supplementary table 6). Next, to investigate the functional roles of transphyletic pCREs, we studied their putative target genes. We found that 110 out of 124 (89%) pCREs were associated with the same conserved genes in all the species. These genes were highly enriched in conserved developmental genes, including multiple TF and signalling pathway gene families, such as Irx, Msx, Pbx, Six4/5, Tbx2/3, Hey, Neurog, Id, Foxn, Smad, Fzd, Bmper and Egfr (Supplementary table 7). Accordingly, the most highly enriched Gene Ontology (GO) terms were related to transcriptional regulation, TFs and cis-regulatory elements, and we obtained almost identical results when using the genes associated with the full set of transphyletic pCREs than when restricting the GO analyses to genes associated with ambulacrarian (i.e. conserved with hemichordates) or with deuterostome (conserved with amphioxus) pCREs (Extended Data Fig. 7c-e Supplementary tables 8-13). Finally, we focused on a core set of 20 conserved elements with shared accessibility across sea star, sea urchin and amphioxus (Extended Data Fig. 7b). We selected one of these 20 pCREs to perform functional assays in sea star and sea urchin embryos, a 5’ proximal element associated with Tbx2/3 genes, a family of TFs highly conserved in animals^85^. This region had been identified as a deeply conserved non-coding sequence in previous works^35,37^ (Fig. 5b,c). We cloned the two orthologous elements from sea star and sea urchin into GFP reporter vectors and assessed their gene regulatory activity by injecting them in embryos of the bat sea star, the purple sea urchin, and a second sea urchin species, *Paracentrotus lividus* (Fig. 5d, Extended Data Fig. 8a,b). The sea urchin Tbx2/3 endogenous spatiotemporal expression profile has been previously characterized and was found to be transiently expressed in several embryonic and larval domains. This includes both the skeletogenic and non-skeletogenic mesenchyme, dorsal regions of the gut, the anterior neuroectoderm, and the aboral/dorsal ectoderm, a domain in which it acts as an early activator of the respective GRN^86–88^. Similarly, in sea star embryos, Tbx2/3 was previously found to be expressed in equivalent domains with the exception of the mesoderm^89,90,91^ (Extended Data Fig. 8c). The GFP expression driven by the sea star and sea urchin Tbx2/3 pCREs in the two species of sea urchin embryos (Fig. 5d) matched the embryonic territories where the endogenous *Tbx2/3* gene is also expressed. Similarly, in sea star embryos injected with either sea urchin or sea star constructs, GFP expression was also consistent with the endogenous *P. miniata Tbx2/3* expression (Fig. 5d). Thus, both the sea urchin and sea star Tbx2/3 CREs are able to drive the same expression patterns independent of the species of origin. Noteworthy, our results also indicate that Tbx2/3 expression in the aboral/dorsal ectoderm and the archenteron is conserved between these two distant echinoderm lineages (Fig. 5e). This showed that this ancient Tbx2/3 CRE is also conserved at the functional level.

## Discussion

By studying 3D chromatin organization and accessibility in echinoderms, we have deepened our understanding of the evolution of animal regulatory genome organization.

Our results added echinoderms to the growing list of animals with TAD-like domains^18,22,58–65^, supporting the view that these 3D structures are ancestral to animals. This conclusion is further reinforced by the close association we and others observed between conservation of ancestral microsyntenic blocks and TADs^3,14,15,17^. From a mechanistic perspective, CTCF motifs were enriched at echinoderm TAD boundaries and in non-dynamic pCREs, suggesting that CTCF contributed to chromatin architecture in the bilaterian ancestor. However, the nature and relative importance of this contribution is uncertain. The absence of CTCF motif orientation patterns in echinoderms contrasts with the orientation-dependent loop organization characteristic of vertebrates^19–22,92–94^ and resembles more the situation in fruit flies, where CTCF function is orientation-independent and less relevant^24^. These findings suggest that the vertebrate mechanism of CTCF-mediated loop formation is derived rather than ancestral. This view is supported by the presence of long-range 3D chromatin interactions in non-bilaterian animals that diverged before the evolution of CTCF, indicating that this protein was not involved in the origin of distal regulation in animals^18^. Alternatively, the derived features of echinoderm and other ambulacrarian CTCFs, including the loss of ancestral Zn-finger domains and high evolutionary rates, could indicate independent losses of an ancestral vertebrate-like state in flies and echinoderms. Resolving these alternative scenarios will require more data from additional bilaterian lineages, including those where CTCF motif orientation has not been conclusively investigated^61,62^.

Patterns of regulatory evolution across echinoderms have renewed long-standing debates about CRE conservation. Most echinoderm pCREs evolve rapidly: about half showed no detectable conservation, and most of the rest were conserved only with closely related species. Such high CRE turnover appears to be a general property of cis-regulatory evolution across animals^27,31–34,69,95–99^. In contrast, the identification of thousands of deeply conserved pCREs—from 1,917 in sea urchin to 9,249 in sea star—was striking. These elements, dating back at least to the late Triassic or even the Permian (200–265 mya), challenge the view that the abundance of ancient regulatory sequences is unique to vertebrates and instead suggest this pattern of regulatory evolution is ancestral to deuterostomes.

A small fraction of elements, a few hundred pCREs, was conserved even deeper, between echinoderm classes or even across deuterostome phyla. Finding similar numbers of transclass and transphyletic pCREs suggests that cis-regulatory sequences across echinoderm classes have diverged almost as much as between deuterostome phyla. This was also reflected in pronounced differences in TFBM usage between sea star and sea urchin development. Nevertheless, the extreme sequence and chromatin accessibility conservation of transphyletic elements—exemplified by the functionally validated Tbx2/3 enhancer—suggest that they regulate core developmental programs shared among deuterostomes. Accordingly, conserved pCREs were particularly enriched at late gastrula, consistent with the higher morphological and gene expression conservation of the mid-developmental stages of multiple animal lineages^7,12,100–104^. In deuterostomes, this period is also marked by a peak in active demethylation of developmental enhancers by TET enzymes^105^.

Low numbers of “Cambrian-age” transphyletic conserved non-coding elements have also been identified in some species of molluscs, brachiopods, chelicerates, priapulids and cnidarians^35–37^ that showed typical hallmarks of slow genome evolution^13,16,46,47^. Although transphyletic elements constitute a tiny fraction of all CREs, our results show they probably indicate that deeply conserved CREs are also widespread in these animals, as suggested by recent results on non-coding conservation in these lineages^106^. If confirmed as CREs by functional genomics data, these conserved elements would indicate that the long-term action of extreme regulatory sequence constraints is an ancestral property of animal genomes. Understanding why certain CREs need to be strongly conserved while most others are easily altered remains a mystery. The identification of deeply conserved CREs in echinoderms (and potentially in other groups) open new paths to solve it. For instance, future studies using scATAC-seq could reveal how these elements are positioned within developmental GRNs. In turn, this will also help to explain why some lineages such as flies and nematodes do not need this type of extreme conservation. This will pave the way to finally understand the so far elusive evolutionary and functional causes underlying CRE sequence conservation.

## Methods

### Animals

*S. purpuratus* and *P. miniata* specimens were provided by Patrick Leahy (Kerckhoff Marine Laboratory, California Institute of Technology, Pasadena, CA, USA) and kept in a closed tank system with circulating diluted Mediterranean seawater at 36 ppt and 15ºC. *P. lividus* specimens were collected from the Gulf of Naples and maintained in circulating Mediterranean seawater at 37.8 ppt and 18 ºC. Sea urchin gametes were obtained by vigorously shaking the adults. Eggs were collected by placing the spawning female, aboral side up, over a beaker with filtered seawater (fSW) . Sperm was collected by pipetting from the surface of the spawning male. Gametes of sea star were obtained by making a V-shaped surgical incision on the aboral side of the animal next to the gonads. Gonads were torn up under the dissecting microscope to release gametes. Sea star oocytes were also treated with 10 µM 1-methyladenine after passing through a filter to mature, until the germinal vesicle disappears, after which eggs were washed with fSW. Embryos were cultured in fSW at 15°C or 18°C until the desired stage.

### Genome Sequencing and Contig Draft Assembly

*P. miniata* genomic DNA (gDNA) was obtained anew from the gametes of a single male animal, while *S. purpuratus* was sequenced using the preserved gDNA samples from the same single male preparation used by the Sea Urchin Genome Sequencing Consortium for the first draft of the purple sea urchin genome assembly^46^. High molecular weight (HMW) DNA larger than 50 kbp was selected using the Circulomics Nanobind CBB Big DNA Kit. The isolated DNA was approximately 300 ng/ul and the majority was between 48 kbp and 145 kbp, as verified by a clamped homogeneous electric field (CHEF) gel.

Duke University School of Medicine Sequencing and Genomic Technologies facilities performed large insert library prep using approximately 30 µg of the HMW DNA from each species and PacBio Sequel sequencing with 16 Single Molecule Real Time (SMRT) cells.

The PacBioreads were assembled into contigs using Canu v1.8 for *S. purpuratus*, and Canu v. DEC-2019 for *P. miniata*^107^ with default parameters. We performed Benchmarking Universal Single-Copy Ortholog (BUSCO)^108^ analysis for both species using a set of 978 metazoan genes to estimate completeness of the draft assemblies.

### HiC Library Preparation and Sequencing

*P. miniata* embryos were fixed in 2% paraformaldehyde in phosphate-buffered saline **(**PFA/PBS) at 68 hpf. *S. purpuratus* embryos were fixed in 2% PFA/PBS at 50 hpf.After 10 minutes fixation was quenched by adding glycine to a final concentration of 0.125 M. Subsequently embryos were washed twice with 1x PBS, frozen in liquid nitrogen and sent to the Gómez-Skarmeta lab for HiC library preparation and sequencing. HiC was performed in duplicates for *P. miniata* and *S. purpuratus*, with about 10 million cells per replicate. HiC preparation was based on^19^ with minor modifications, described in detail in^22^. Briefly, nuclei were isolated and permeabilized by SDS treatment. Chromatin was fragmented using the DpnII restriction enzyme (NEB, R0543). Restriction overhangs were blunted and marked with biotin-14-dATP and subsequent proximity ligation was performed in intact nuclei. Purified and sonicated biotin-labeled DNA fragments were enriched using Dynabeads My One C1 Streptavidin beads. Final libraries for paired-end sequencing were prepared using NEBNext High-Fidelity 2X PCR Master Mix (NEB), using TruSeq Primer 1.0 (P5) and TruSeq Primer 2.0 (P7). At least eight independent PCR reactions were performed per replicate and then pooled to maintain library complexity. Libraries were multiplexed and sequenced using DNBseq technology (BGI, China) to produce 50 bp paired-end (PE) reads and approximately 400 million raw sequencing read pairs per sample.

### HiC data analyses

HiC reads from biological replicates were pooled and mapped to the *P. miniata* and *S. purpuratus* newly generated genome assemblies using BWA v0.7.17^109^. Published HiC data for *A. amurensis* (SRR24835318) were mapped to its genome assembly GCA_032118995.1. Ligation events (HiC pairs) were detected and sorted, and PCR duplicates were removed using pairtools (https://github.com/mirnylab/pairtools). Unligated and self-ligated events were filtered out by removing contacts mapping to the same or adjacent restriction fragments. The resulting filtered pairs were input into Juicer Tools 1.13.02 Pre^110^, which generated multiresolution hic files via custom scripts (https://gitlab.com/rdacemel/hic_ctcf-null )^24^: the hic_pipe.py script was first used to generate tsv files with the filtered pairs, and the filt2hic.sh script was then used to generate Juicer hic files. Knight-Ruiz (KR) normalized HiC matrices at 10 Kb resolution^111^, were extracted for downstream analysis (insulation scores, TAD boundaries, aggregate TADs and loops) performed and visualized using the FAN-C 0.9.14 toolkit^112^.

TAD boundaries were called using the insulation score method^113^. Insulation scores were calculated for 10-Kb binned HiC matrices using FAN-C^112^. Briefly, the average number of interactions of each bin were calculated in 250-Kb square sliding windows (25 × 25 bins); then, these values were normalized as the log2 ratio of each bin’s value and the mean of all bins to obtain the insulation score for each bin; next, minima along the insulation score vector were calculated using a delta vector of +/−50 Kb (+/−5 bins) around the central bin. Only boundaries with a score lower than 1.5 were considered, to avoid calling low mappable regions as TAD boundaries. The genomic fragments limited by adjacent boundaries were considered as TADs, excluding fragments limited by a boundary and the start/end of the chromosomes. Ten sets of shuffled TAD boundaries were generated by partitioning the genome into virtual TADs with the same size as experimental ones but randomly positioned within chromosomes, and the median signal of CTCF motif number for each set was calculated for plotting purposes. Chromatin loops were called using Juicer Tools 1.13.02 Hiccups^19^, with standard parameters. Briefly, the multiresolution hic file was used as input for the CPU version of HICCUPS, which ran using 5, 10 and 25-Kb resolution KR-normalized matrices. The maximum permitted FDR value was 0.1 for the three resolutions; the peak widths were 4, 2 and 1 bin for 5, 10 and 25-Kb resolutions, respectively; and the window widths to define the local neighborhoods used as background were 7, 5 and 3 bins, respectively. The thresholds for merging loop lists from different resolutions were the following: maximum sum of FDR values of 0.02 for the horizontal, vertical, donut and lower-left neighborhoods; minimum enrichment of 1.5 for the horizontal and vertical neighborhoods; minimum enrichment of 1.75 for the donut and bottom-left neighborhoods; minimum enrichment of 2 for either the donut or the bottom-left neighborhoods. The distances used to merge the nearby pixels to a centroid were 20, 20 and 50-Kb for 5, 10 and 25-Kb resolutions, respectively. For *P. miniata* and *S. purpuratus*, only loops whose anchors were located in the same contig were considered, to ensure that the identified loops were not affected by any putative undetected misassembly. This was not applied in the case of *A. amurensis* because there was no publicly available information on the contig structure of the published GCA_032118995.1 genome assembly and in *D. melanogaster*, where its chromosome level genome assembly has been extensively curated. Ten sets of shuffled loops were generated so that they kept the same distances between anchors as those of experimental ones but randomly positioned within chromosomes, and the median signal of CTCF motif number for each set was calculated and plotted.

### Creating Scaffolds

The draft contigs for each species were assembled into scaffolds using the 3D de novo assembly (3D-DNA) v.180922 pipeline (https://github.com/aidenlab/3d-dna)^114^ and visualized with Juicebox (https://github.com/aidenlab/Juicebox)^115^. The draft contigs were assembled into scaffolds with the 3D-DNA *run-liger-scaffolder* program, using contigs larger than 6 kbp and HiC reads with a MAPQ alignment score greater than 0. HiC contact heatmap revealed a large number of overlapping contigs,these were dealt with using the 3D-DNA *align-nearby-sequences-and-filter-overlaps* and *tile-assembly-based-on-overlaps* programs. This reduced the size of the draft assembly from 1075 Mbp to 714Mbp. The finalized assemblies were submitted to NCBI GenBank: GCA_015706575.1 (*P. miniata* v3.0) and GCA_000002235.4 (*S. purpuratus* v5.0).

### Annotation of the assemblies

Assemblies were annotated using the NCBI eukaryotic genome annotation pipeline^57^ using publicly available Entrez transcripts and RNA-seq reads from the Sequence Read Archive (SRA). Gene content was also evaluated by using BLAST^116^ to align the previous assembly’s gene models to the new assembly.

### Generation of cohesin (SA1–3) and CTCF gene trees

Protein sequences of cohesin subunit SA1–3 and of CTCF were searched using the NCBI Blast, against Refseq genomes and proteomes and applying the “taxonomy” filter to search of relevant echinoderm outgroups such as hemichordates *Mus musculus* and *Drosophila melanogaster* protein sequences were used as initial queries. Then, a second search was performed using the retrieved protein hits as new queries. *Hemicentrotus pulcherrimus* SA1–3 protein sequence was obtained from transcriptome shotgun assembly data.

For the phylogenetic analyses, protein sequences were aligned with the MAFFT v7.505 software^117^ and the resulting alignments were trimmed (Supplementary Data 1,2) using Aliview v1.28 to discard spuriously aligned regions^118^. Phylogenetic trees were built using IQ-Tree 2 v2.0.7^119^, and tested with UFBoot (bootstrap = 10^3^)^120^ and an approximate Bayes test for single branch testing. Following previous CTCF phylogenetic studies^121^, the tree was rooted using the CROL proteins of *D. melanogaster* and *Tribolium castaneum*. In SA1–3 proteins, the tree was rooted using orthologs from sponges. The model used (LG+G4+I for both trees) was selected using ModelFinder with BIC^122^. Trees (Supplementary Data 3,4) were visualized using ITOL v7^123^.

### Multiple Genome Alignments

A multiple genome alignment was constructed using Progressive Cactus v1.2.3^124^ with default parameters. This contained our two newly reported genomes, a broader sampling of echinoderms, and two marine deuterostomes. A full list of included species and genome accession numbers can be found in Extended Data Fig 6a. Then, the genomes were masked for repeats using RepeatMasker v4.1.1 with-pa 8; -xsmall parameters. Finally, we provided the species tree in Newick format. The resulting .hal file and associated .hub files are hosted on the Echinobase^125^ FTP server (https://download.xenbase.org/echinobase/Genomics/user-submitted/MGA_echinoderms/NewMGA/FinalMGA/) and Figshare (doi.org/10.6084/m9.figshare.30506378).

### ATAC-seq library preparation

ATAC-seq libraries were generated as described in^126,127^. Cultured embryos were collected into tubes to obtain ~50,000 cells, then centrifuged at 500 x g, and washed with fASW twice. The embryos were then lysed in 50 μl of lysis buffer (10 mM Tris(hydroxymethyl)aminomethane pH 7.4, 10 mM NaCl, 3 mM MgCl_2_ and 0.2% NP40/Igepal CA-630). The half of nuclei suspensions were incubated for 30 minutes at 37°C with 25 μl of 2x tagmented DNA buffer (TD buffer: 20 mM Tris; 10 mM MgCl_2_; 20% v/v dimethylformamide) and 1.25 μl Tn5 enzyme and Tagmentation Buffer (10 mM Tris pH 8.0, 5 mM MgCl_2_, 10% w/v dimethylformamide), and incubated for 30 min at 37ºC. Then, tagmented DNA was purified with the Qiagen MinElute kit (Qiagen, 28004) and eluted in 10 μl. PCR reactions for each replicate were performed with 15 cycles using Ad1F and different AdR primers^126^ and NEBNext High-Fidelity 2X PCR Master Mix (New England Labs Cat #M0541). The resulting libraries were multiplexed and sequenced on the HiSeq 4000 sequencer.

### ATAC-seq peak calling and genomic distribution

Data analysis for ATAC-seq libraries were performed as described in^127^. After sequencing, paired-end reads without adapter sequences were aligned against the reference genome using Bowtie2 v2.2.6^128^ with *-X 2000 -no-mixed -no-unal* parameters. PCR artifacts and duplicates were removed using Samtools v1.3 *rmdup* ^129^. Read start sites were offset by +4 or by −5 bp in the plus or minus strand, respectively. Nucleosome-free pairs (insert < 130 bp) retained for peak-calling using MACS2 v2.1.1.20160309^130^ with the following parameters: *–nomodel –shift −45 –extsize 100* and the genome size of the correspondent organism. The irreducible discovery rate (IDR v2.0.3^131^) was used to assess replicability with: *–input-file-type narrowpeak –rank p.value –soft-idr-threshold 0.1* and the MACS2 peak calling parameters. Called IDR peaks were split into promoter, gene body and noncoding regions using the aforementioned gene annotation. ATAC peaks were defined as ”promoters” if they overlapped an annotated gene TSS; as “proximal” if located in noncoding regions within 5-kbp upstream of a TSS but without overlapping with it, and as “distal” if they were located in other noncoding locations not included in the previous categories.

### ATAC-seq differential analyses and TFBM enrichments

The number of reads inside IDR peaks peaks were calculated with intersectBed from BEDtools v2.26.0^132^, employing the *-c* parameter, IDR peaks as reference and filtered reads as query. The resulting count table was used in DESeq2 v1.18.0 R package^133^ to get developmental dynamic peaks (differentially accessible peaks with p value<0.05)s. Peaks without significant accessibility changes were considered non-dynamic. In the case of *S. purpuratus*, differential analyses were performed with two different sets of merged IDR peaks, one obtained with the full set of *S. purpuratus* stages, to identify all dynamic peaks in this species, and a second one with 35,831 IDR from developmental stages matching the *P. miniata* samples. Peaks were associated with their putative target genes using BEDtools closestBED, with the parameters *-D ref -iu -no-namecheck -k 1*. In order to find TFBM enrichments in ATAC peaks the script FindMotifsGenome.pl from HOMER v3.12 software^134^ was used with *–mset* parameter and using merged IDR peaks as background model. To find specific temporal dynamics in open chromatin regions, dynamic peaks from second peakset analyses were analyzed with seqMINER v1.3.4^135^ and cluster heat map plots were generated by computeMatrix deepTool v3.4.3^136^.

### Evolutionary conservation of pCREs

To assess the evolutionary conservation of pCREs only non-exonic peaks lacking hits in a Blastx analysis were considered, to avoid false positives of conserved coding sequences. The resulting set of ATAC-seq in the query species were then lifted over to the target species using halLiftover v2.1^137^ with the option --noDupes. When ATAC-seq regions in the target species were not available, only sequence conservation was assessed. Whenever ATAC-seq information was available in the target species (that is, in *S. purpuratus*, *P. miniata* and *B. lanceolatum*), we retrieved the correspondence between ATAC-seq peaks in both query and target species. Liftovered peaks were intersected with BEDtools (options -f 0.5 -r or -f 0.5 -r -a) with the target species ATAC-seq peaks, resulting in set of reciprocal ATAC-seq peaks had at least 50bp overlapping with the original ATAC peak coordinate of the query species. Finally, resulting files were grouped following their relative evolutionary distance (i.e. the different strata) from the query species by using subset() and merge() R basic command (https://www.R-project.org/). Conserved regions belonging to the most ancient strata (echinoderms and deuterostomes) were independently retrieved using *S. purpuratus* and *P. miniata* ATAC peaks as queries. Therefore, the non-redundant sets were obtained by checking if any of the genomic coordinates identified by *P. miniata* peaks in the different target species, *Ap. japonicus*, *An. japonica*, *S. kowalevskii* and *B. lancelolatum* overlapped with the coordinates retrieved through *S. purpuratus* peaks or corresponded to *S. purpuratus* peaks in the *P. miniata* - *S.purpuratus* alignment. To analyze conserved accessibility in transphyletic pCREs, all cases of pCREs conserved at the sequence level with another species where the conserved orthologous region did not have conserved accessibility were manually inspected in the genome browser. This identified false negative cases that have been filtered out in the target species because the corresponding ATAC-peak partially overlapped with an annotated exon. In most cases, these exons only overlapped with a small part of the ATAC peak or the portion that overlapped corresponded to a UTR, and we were able to verify that the blocks of conserved sequence we have detected did not overlap with any coding exon. This recovered 40 regions with conserved accessibility (labelled as PEpeak, for Partially Exonic peak in Supplementary Table 6), and discarded 11 regions that did actually overlap with coding exons missed by our prior filters(unannotated exons in the query species and small/fast evolving exons not detectable by Blastx searches), resulting in a final set of 134 conserved transphyletic pCREs.

### Identification of conserved target genes of transphyletic pCREs

To investigate conservation of transphyletic pCREs associated genes, gene orthogroups (Supplementary Data 5) were inferred with Orthofinder v2.5.5^138^, using DIAMOND v0.9.19.120^139^ as alignment tool and manually providing the species tree of the following species, for which the longest coding isoform of eahc gene was extracted: *B. lanceolatum* (BraLan2), *Callorhinchus milii* (IMCB_Cmil_1.0), *Homo sapiens* (GRCh38.p14), *Monodelphis domestica* (MonDom1), *Xenopus tropicalis* (GCF_000004195.4), *D. rerio* (GRCz11), *Lepisosteus oculatus* (fLepOcu1.hap2), *S. purpuratus* (Spur_5.0), *P. miniata* (Pmin_3.0), *S. kowalevskii* (Skow_1.1) and *N. vectensis* (jaNemVect1.1). For each accessible peak in *B. lanceolatum*, *S. kowalevskii*, *S. purpuratus* and *P. miniata*, the two closest flanking genes included within an orthogroup, were identified using the closestBED tool utility from BEDtools v2.31.0, with the parameters -D a -id (or -iu) -k 1. All overlapping genes were kept if more than one overlapped the accessible peak region. All pCREs where at least one of the two closest genes belonged to the same orthogroup across the different species were considered as associated to the same conserved genes. All non-matching cases were manually curated to identify potential false negative cases of conserved genes (i.e. unannotated genes or artefactual gene models not correctly assigned to orthogroups). In 10 of the 134 pCREs gene conservation could not be assigned because in one species the pCRE was located near the end of agenomic scaffold or because the pCREs were assigned to different orthogroups that contained multiple paralogous genes whose orthology could not be clearly established. Furthermore, for two ambulacrarian regions, conservation of associated genes could not be determined *S. kowalevskii*, and therefore were provisionally assigned to the Echinodermata stratum (Supplementary Table 7).

### Gene Ontology Enrichment

A new GO annotation was built de novo by running the software FANTASIA v1^140^ on the *S. purpuratus* proteome, including only the longest isoform of each protein-coding gene. This GO annotation was then transferred to the corresponding orthogroups, collapsing the orthologues associated terms and discarding the redundant terms. The GO enrichment analysis was performed using the topGO v2.56.0 R package^141^ with a Fisher’s exact test for Biological Process and Molecular Function separately. Significantly enriched terms were defined as those with p_adj_ < 0.05. Term definitions were retrieved using the GO.db.

### CRE DNA tag synthesis

Tagging of putative CREs was performed as in^70^. Primers for the putative CREs (Supplementary Table 14) were designed using Primer3web v4.1.0^142^ with additional 18bp of the reverse complement of the beginning of the DNA tag sequence added to the 5’ of the reverse primer to ensure that the CRE can be combined with the DNA^143^. Equal amounts of CRE and Tag DNA were combined using overlap PCR^70,143^ using Expand High Fidelity PLUS PCR (Sigma). The product was gel purified using 2% agarose 1X TAE gel and GenElute Gel Extraction Kit (Sigma) (FoxA pCREs) or NucleoSpin Gel & PCR Clean-up (Macherey-Nagel) (Tbx2/3 pCREs) according to manufacturer’s guidelines and eluted in 50 μl of Elution buffer. The eluted DNA was then purified again using QIAquick PCR Purification Kit (Qiagen) and eluted in 30 μl of elution buffer. NanoDrop ND-1000 (ThermoFisher Scientific) was used to assess DNA yield and quality.

### CRE Microinjections

Sea urchin eggs were de-jellied and placed on 1% protamine sulfate (PS) treated plates and then fertilized, while the sea star ones placed on the PS-plates after fertilization, and were injected with 2 pL and 8 pL of microinjection solution, respectively. Microinjection solutions consisted of 0.5 μl of tagged CRE pool, 1.2 μl of 1mM KCl, 0.275 μl of 500 ng/μl carrier DNA and water up to 10 μl^70,144^. Injected eggs were then washed twice with fSW and incubated at 15°C for *S. purpuratus* and *P. miniata* and at 18°C for *P. lividus*. At least two independent batches of embryos were injected and at least 30 embryos per experiment were analyzed.

### Genomic DNA and mRNA extraction from microinjected embryos

Microinjected embryos of the desired stage were collected into 1.5 ml tubes (Eppendorf). AllPrep DNA/RNA Micro kit (Qiagen) kit was used to extract genomic DNA and RNA from them according to manufacturer’s guidelines. DNA and RNA were eluted in 100 μl and 18 μl of 65°C nuclease free water, respectively. The RNA was treated with DNase from RNAqueous-Micro Total RNA Isolation Kit (Invitrogen) according to manufacturer’s guidelines. 14 μl of the DNase treated RNA was used to synthesize cDNA using SuperScript VILO cDNA Synthesis Kit (Invitrogen) as per manufacturer’s guidelines. The synthesized cDNA and eluted DNA were used for qPCR quantification.

### qPCR quantification of CRE expression

Extracted gDNA and synthesized cDNA were used to estimate relative expression of the microinjected tagged CREs in two biological replicates. Prior to qPCR, the cDNA was amplified using universal primers^70^ using the following thermocycler program: 2 minutes at 95°C, 21 cycles of 15 seconds at 95°C, 30 seconds at 60°C and 1 minute at 72°C, followed by 5 minutes at 72°C and hold at 4°C. The product was purified using QIAquick PCR Purification Kit (Qiagen) and eluted in 30 μl and used for qPCR quantification: 5 μl of Fast SYBR Green Master Mix, 4 μl of 0.7 pmol/ul qPCR primers and 1 μl of cDNA/gDNA using ViiA7 Real–Time PCR System machine (Life Technologies) and 20 seconds at 95°C, 40 cycles of 1 second at 95°C and 20 seconds at 60°C, followed by melting curve at 95°C for 15 seconds, 60 °C for 1 minute and 95°C for 15 seconds program. Total GFP expression was used as control for quantification of cDNA and genomic DNA with the same specific tag primers. The number of tags expressed was normalized to the number of tags in the gDNA by dividing the number of expressed tags in cDNA by the number of expressed tags in gDNA relative to GFP. Mean of the between-replicate values was used for plotting.

### Fluorescent in situ hybridization (FISH)

The primes used to isolate the Sp-Tbx2/3 cDNA from gastrula stage total cDNA are described in Valencia et al., 2021. Digoxigenin-labelled antisense RNA probe for Sp-Tbx2/3 was synthesized as previously shown^145^. *S. purpuratus* gastrula stage embryos (48 hpf) were collected and fixed for 1h at RT in 4% PFA in MOPS buffer (0.1 M MOPS pH 7, 0.5 M NaCl and 0.1% Tween-20 in nuclease-free water). Embryos were washed several times with MOPS buffer and stored in 70% ethanol at −20°C. After rehydration, embryos were subjected to FISH as previously described^146^.

### Imaging

pCRE microinjected and FISH embryos were imaged using GFP and bright-field settings^144^ using Axio Imager.Z2 (Zeiss) and LSM 700 (Zeiss) confocal microscope, respectively. Images were processed using ImageJ v1.52o.

## Extended Data

## Supplementary Material

SData1

SData2

SData4

SData3

SData5

Supp Tables All

## Data Availability

Genome assemblies used in this work are publicly available at the NCBI genome database GenBank with accession numbers GCA_015706575.1 (*P. miniata*) and GCA_000002235.4 (*S. purpuratus*). The HiC (GSE281901) and ATAC-seq (GSE280529) raw and processed sequencing data were deposited at the Gene Expression Omnibus (GEO) database under accession code of the SuperSeries GSE281904, with the exception of ATAC-seq data in *S. purpuratus* at 48 hpf that were deposited in GSE186363. Files and share links for viewing the multiple genome alignment are available in Figshare (doi.org/10.6084/m9.figshare.30506378)^147^.
